# Molecular evaluation and phenotypic screening of brown and orange rust in *Saccharum* germplasm

**DOI:** 10.1371/journal.pone.0307935

**Published:** 2024-07-30

**Authors:** Gleicy Kelly Oliveira, Fernanda Zatti Barreto, Thiago Willian Almeida Balsalobre, Roberto Giacomini Chapola, Hermann Paulo Hoffmann, Monalisa Sampaio Carneiro

**Affiliations:** 1 Department of Biotechnology, Vegetal and Animal Production, Federal University of São Carlos, Araras, SP, Brazil; 2 Sugarcane Breeding Program of RIDESA/UFSCar, Araras, SP, Brazil; North Dakota State University, UNITED STATES OF AMERICA

## Abstract

Brazil is the largest global producer of sugarcane and plays a significant role—supplier of sugar and bioethanol. However, diseases such as brown and orange rust cause substantial yield reductions and economic losses, due decrease photosynthesis and biomass in susceptible cultivars. Molecular markers associated with resistance genes, such as *Bru1* (brown rust) and *G1* (orange rust), could aid in predicting resistant genotypes. In this study, we sought to associate the phenotypic response of 300 sugarcane accessions with the genotypic response of *Bru1* and *G1* markers. The field trials were conducted in a randomized block design, and five six-month-old plants per plot were evaluated under natural disease conditions. Genotypic information about the presence or absence of *Bru1* (haplotype 1) and *G1* gene was obtained after extraction of genomic DNA and conventional PCR. Of the total accessions evaluated, 60.3% (181) showed resistance to brown rust in the field, and of these, 70.7% (128) had the *Bru1* gene present. Considering the field-resistant accessions obtained from Brazilian breeding programs (116), the *Bru1* was present in 77,6% of these accessions. While alternative resistance sources may exist, *Bru1* likely confers enduring genetic resistance in current Brazilian cultivars. Regarding the phenotypic reaction to orange rust, the majority of accessions, 96.3% (288), were field resistant, and of these, 52.7% (152) carried the *G1* marker. Although less efficient for predicting resistance when compared to *Bru1*, the *G1* marker could be part of a quantitative approach when new orange rust resistance genes are described. Therefore, these findings showed the importance of *Bru1* molecular markers for the early selection of resistant genotypes to brown rust by genetic breeding programs.

## Introduction

Sugarcane (*Saccharum* spp.) is an essential crop for sugar production, accounting for 80% of global sugar consumption [1]. It is also one of the primary sources of first- and second-generation ethanol, a sustainable alternative with high potential to mitigate the effects of climate change without affecting food security [2,3]. Additionally, sugarcane generates other byproducts, such as biodiesel, bioelectricity, bioplastics, and fertilizers [4]. It is cultivated on 26 million hectares worldwide, and approximately 1.9 billion tons of sugarcane are produced annually [1]. Brazil is the largest producer, with approximately 35% of the world’s planted area, production estimated at 598.3 million tons of sugarcane in the 2022/2023 harvest, and productivity estimated at 72 t/ha [5].

To obtain a new sugarcane cultivar, after the hybridization stage, many clones are commonly evaluated during several harvests in multi-environment trials. Furthermore, several traits are evaluated simultaneously such as yield, resistance to diseases, pests and abiotic stresses. So, sugarcane breeding programs seek to obtain new cultivars adapted to different growing conditions, maximizing the presence of desirable traits, in a process that can take 10 to 15 years [6–8]. In addition to the challenges of agronomic and management practices, the genetic complexity of modern sugarcane cultivars due to the high number of chromosomes (ranging from 100 to 130), a large genome (>10 Gb), and the occurrence of aneuploidy, with a variable number of chromosomes in each homology group [9–13], makes it difficult to assemble all favorable alleles into a highly heterozygous outcrossing plant [6–8]. This complexity also hampers the integration of quantitative traits and molecular data, consenquetely, to understand the genetic architecture responsible for increasing yields. Conventional breeding of sugarcane in Brazil has consistently been one of the major contributors to improving agroindustrial yield over the last decades and providing security against outbreaks of pests and diseases. However, due to the high genetic complexity of the crop, low heritability in the strict sense of most economically important traits, and the lengthy breeding cycle, detailed knowledge of quantitative genetics and possibly new innovative breeding strategies are needed to continue genetic improvement [6].

Varietal resistance is the most viable control strategy for the leading diseases in sugarcane. Among the foliar diseases of sugarcane, two species of *Puccinia* fungus cause brown rust (*P*. *melanocephala* Syd. & P.Syd) and orange rust (*P*. *kuehnii*) [14]. The progression of brown and orange rust depends on primary factors such as host genotype, environmental conditions (temperature and humidity), and inoculum pressure [15,16], and the spores are easily spread by wind and rain. In susceptible cultivars, the pathogen impairs the photosynthetic rate and related pathways, causing a reduction in the height and diameter of the stalks, reducing the number of stalks, and compromising the final production of biomass [15,17,18]. The chlorosis resulting from leaf pathogen infection is linked to chlorophyll loss. Fungi causing necrotic leaf reactions often affect leaf photosynthesis. Decreases in physiological traits, such as reduced green leaf area index and functional chloroplast decline leading to reduced photosynthetic rates, have been associated with crop yield losses [18].

Brown rust is present in almost all cultivation areas [19–21]. It is estimated that losses of 10–20 tons of sugarcane per hectare depend on the time that the fungus affects the crop [18,22], potentially reaching losses of 50% in production [23]. Orange rust occurs in approximately 45 sugarcane-producing countries [16]. Losses of 30 to 50% have been recorded in Australia, Brazil, and the USA when varieties susceptible to orange rust were used [22,23].

Globally, sugarcane genetic improvement programs have been working to develop varieties resistant to brown and orange rust [6,16,24]. For example, sugarcane varieties resistant to brown rust (*P*. *melanocephala*) were developed using the major rust resistance gene *Bru1*, which was identified in cultivar R570 [19,25]. Additionally, for brown rust, there are reports of alternative sources of resistance [26–30]. Concomitantly, molecular markers, such as the *Bru1* marker [29,30] for brown rust and the *G1* marker [31] for orange rust, have been proposed to predict resistance in modern sugarcane cultivars. Similarly, molecular markers associated with genes responsible for resistance to these diseases can help breeding programs confirm the introgression of favourable alleles, find new sources of resistance, and release new cultivars with durable resistance [32–34]. Therefore, using a core collection of 300 sugarcane genotypes, the objectives of this study were: (i) field evaluation and genotyping of molecular markers linked to resistance to brown (*Bru1*) and orange (*G1*) rust; (ii) to evaluate markers for predicting the resistance/susceptible phenotype and their potential application in marker-assisted selection (MAS); and (iii) to track the presence/absence of the *Bru1* marker in the genealogy of a modern sugarcane variety widely cultivated in Brazil.

## Materials and methods

### Plant material

Molecular analyses (using *Bru1* and *G1* markers) and field phenotypic evaluations of brown and orange rust were conducted on a nuclear collection comprising 300 sugarcane accessions (see S1 Table). This collection consisted of 242 accessions from the Brazilian Panel of Sugarcane Genotypes (BPSG) [35,36], nine varieties currently cultivated in commercial areas in Brazil (CTC4, CTC9001, RB975033, RB975201, RB975952, RB975375, RB005014, RB015177, and RB015935) [24], and 49 precommercial sugarcane hybrids from the genetic improvement program of Rede Interuniversitária para o Desenvolvimento do Setor Sucroenergético (RIDESA). It is important to consider that the BPSG brings together the main parents of Brazilian breeding programs in last decades, relevant cultivars from countries where sugarcane is grown, representative genotypes of the *Saccharum* species (*S*. *offcinarum*, *S*. *spontaneum*, *S*. *robustum*, *S*. *sinense*, *S*. *barberi*, *S*. *edule*), *Erianthus* spp. accessions, and important cultivars to Brazilian mapping programs [35,36].

### Evaluation of natural brown and orange rust infection in the field

The experiments were conducted at the Agricultural Sciences Center of the Federal University of São Carlos (UFSCar) in the city of Araras, State of São Paulo, Brazil (22° 21’ 25" S, 47° 23’ 03" W, 629 m a.s.l.). The site is characterized by a latosol, an annual rainfall of 1575 mm. The climate is classified as Cwa mesothermic (Köppen classification) with hot and humid summers and dry winters. The annual average temperature in the experimental area is 21.5°C ranging from 17.9°C in the coldest month (July) to 24.2°C in the hottest month (February) [24]. Historically, this location has experienced natural occurrences of brown rust (*P*. *melanocephala*) and orange rust (*P*. *kuehnii*) due to climatic conditions, particularly between November and March, when the average monthly rainfall and temperature are approximately 173 mm and 23°C, respectively [24,35].

The BPSG accessions were evaluated following a randomized block experimental design with four replications, where each experimental plot consisted of two 3.0 m rows spaced 1.40 m apart [35]. The trial with commercial cultivars and precommercial hybrids followed a randomized block design with two replications, where each experimental plot comprised two 5.0 m rows spaced 1.40 m apart. The commercial cultivars RB867515 and RB966928 were included as standards in the experiments because of their widespread cultivation in Brazil [6].

For each accession, five six-month-old plants (per plot) were evaluated under conditions of natural disease inoculation. The severity of brown rust and orange rust was visually assessed on leaves +3 and +1, respectively, using the diagrammatic scale of Amorim et al. [37]. For brown rust, accessions with a grade of 1 were classified as resistant, while those with scores ranging from 2 to 9 were considered susceptible. Cultivars SP80-3280 and RB835486 were used as resistant and susceptible checks to brown rust, respectively. Regarding orange rust, accessions with scores up to 3 were considered resistant, whereas those with scores ranging from 4 to 9 were considered susceptible. Cultivars SP80-3280 and SP81-3250 were used as resistant and susceptible checks to orange rust, respectively. Examples of the infection brown rust and rust orange are illustrated in S1 Fig.

### *Bru1* and *G1* marker analysis

Genomic DNA was extracted from 3.0 g of leaf primordium tissue following the method described by Aljanabi et al. [38]. The conditions for amplifying the markers R12H16 and 9O20-F4-RsaI associated with *Bru1*, as well as the visualization of the amplified fragments, were based on those of Costet et al. [30]. Briefly, amplification was performed with 50 ng of DNA mixed with 1×PCR buffer, 2 mM MgCl_2_, 0.2 mM dNTP, 0.2 μM forward primer, 0.2 μM reverse primer, 0.5 U DNA polymerase in a final volume of 20 μl. The PCR profile used was: one step of 94°C for 5 min followed by 35 cycles of 94°C for 30 s, 55°C for 30 s, and 72°C for 45 s. Then, followed a final elongation step at 72°C for 5 min. Fifteen microliters of the 9O20-F4 PCR products was digested with 1× NEBuffer1 and 5U RsaI (New England Biolabs). This digestion mix was incubated at 37°C for 2 h. The PCR products of R12H16 and 9O20-F4-RsaI were run on a 2% agarose gel. The accessions were categorized into haplotypes based on the fragments amplified by the molecular markers, with haplotype 1 assigned to accessions presenting fragments from both markers and haplotype 4 assigned to accessions not showing either of the two fragments. Only accessions containing the fragment amplified by the R12H16 marker were classified as haplotype 2, while those containing only the fragment amplified by the 9O20-F4-RsaI marker were classified as haplotype 3 [39]. Amplification of the *G1* marker and visualization of the 950 bp amplified fragment were performed following the protocols outlined by Yang et al. [31] and Fier et al. [32]. Briefly, amplification was performed in 20μl reaction containing 10× PCR buffer (10 mM Tris-HCl, 50 mM KCl), 2.5 mM MgCl_2_, 0.2 mM each dNTP, 1 μM each forward and reverse primer, 0.5U DNA polymerase, 40 ng template DNA and ultrapure water to complete volume. Touchdown PCR was performed; after initial denaturation at 95°C for 5 min, four steps were carry out: i) five cycles of 1 min denaturing at 96°C, 5 min annealing at 68°C with a decrease of 2°C in each subsequent cycle, and 1 min extension at 72°C; ii) five cycles of 1 min denaturing at 96°C, 2 min annealing at 58°C with a decrease of 2°C in each subsequent cycle, 1 min extension at 72°C; iii) 25 cycles of 1 min denaturing at 96°C, 1 min annealing at 50°C and 1 min extension at 72°C; and iv) a final extension at 72°C for 5 min. PCR products were run on 1% agarose gel in horizontal electrophoresis. Genotypes were classified based on the presence (1) or absence (0) of the amplified fragment.

### Frequencies of *Bru1* and *G1* markers in relation to the origin of the accessions and across time

Molecular characterization of the *Bru1* and *G1* markers was conducted on the nuclear collection of 300 accessions, considering the origin of the genotypes. Initially, the accessions were categorized into three groups, as proposed by Medeiros et al. [36] and Barreto et al. [35]: group A comprised ancestral germplasm, group FH included genotypes from foreign breeding programs, and group B consisted of hybrids from Brazilian breeding programs. This third set was further divided into subsets based on the year in which the crossing was conducted to obtain the genotype, namely, B1 (1940–1951), B2 (1952–1961), B3 (1962–1971), B4 (1972–1981), B5 (1982–1991), B6 (1992–2001), and B7 (2002–2011) (refer to S1 Table). The presence of the *Bru1* and *G1* genes was also assessed in a historical series of the ten varieties most planted between 1974 and 2022 in the southcentral region of Brazil (states of São Paulo and Mato Grosso do Sul). This production region accounts for approximately 50% of the sugarcane production area in Brazil [5].

### Genealogical exploration of the *Bru1* gene in modern cultivars

The RB966928 variety [40] is a modern cultivar that had a significant commercial impact and is extensively cultivated in the southcentral region of Brazil, covering approximately 582 thousand hectares [6,41]. The pedigree was constructed using kinship data with PedigraphTM software [42]. Information on the haplotypes of molecular markers associated with *Bru1*, along with the results of phenotypic characterization for brown rust (resistant or susceptible), was incorporated into the genealogy. Additionally, seven accessions (Co285, EK28, CP27-34, CP1165, CP27-139, CP38-34, and CP53-18), which were not originally part of the nuclear collection evaluated in this study, were analysed for the markers R12H16 and 9O20-F4-RsaI following a previously described procedure.

## Results

### Severity and incidence of brown rust in the field

The results of the field phenotypic assessment of brown rust are summarized in Tables 1 and S1. Out of a total of 300 evaluated accessions, 181 accessions (60.3%) were rated as 1 (resistant), while 119 accessions (39.7%) received ratings from 2 to 9 (susceptible), considering the average values of the ratings on the diagrammatic scale obtained from the two years of evaluation. Among the 181 resistant accessions, 50 belonged to ancestral germplasms (A) (Table 1 and S2A Fig). Half of the accessions of *S*. *officinarum* and *S*. *robustum*, as well as all genotypes of *S*. *spontaneum*, *S*. *sinense*, *S*. *barberi*, *S*. *edule*, and *Erianthus*, showed resistance to brown rust. The hybrid Badila and genotypes from India, such as Chin, Chunnee, Ganda Cheni, Maneria, SES 205A, White Mauritius, White Pararia, and White Transparent, were also resistant (S1 Table). A total of 15 foreign accessions (FHs) (41.6%) were resistant to brown rust (Table 1 and S2A Fig). The FH resistant group included genotypes from POJ2878 and R570. Australian accessions (Q70, Q165, and Q117) and Argentinean accessions (NA56-79 and TUC71-7) were susceptible to brown rust under field conditions (S1 Table).

**Table 1:** Field evaluation of brown rust reaction and genotyping of molecular markers for the *Bru1* gene in a nuclear collection of 300 sugarcane accessions ancestral germplasm (A), foreign hybrids (FH), and Brazilian breeding (B)^2^.

	Resistant^3^					Susceptible^3^				
Group	Haplotypes^1^					Haplotypes^1^				
	**1**	**2**	**3**	**4**	**Total**	**1**	**2**	**3**	**4**	**Total**
**Ancestral access (A)**	23	9	2	16	50	0	5	4	17	26
**Foreign hybrids (FH)**	15	0	0	0	15	0	4	1	16	21
**Brazilian Breeding Programs (B)^2^**										
**RB**	71	9	9	5	94	0	8	0	29	37
**SP**	11	1	0	0	12	0	10	0	8	18
**CTC**	1	0	0	0	1	0	0	0	1	1
**IAC**	4	0	1	0	5	0	3	0	5	8
**CB**	3	0	1	0	4	0	3	0	6	9
**Total**	128	19	13	21	181	0	33	5	81	119

^1^ *Bru1* gene based on the molecular markers R12H16 and 9O20-F4-RsaI genotyping as described by Costet et al., [30].

^2^ Brazilian Breeding Programs: RB: Rede Interuniversitária para o Desenvolvimento do Setor Sucroenergético; SP: Copersucar; CTC: Centro de Tecnologia Canavieira; CB: Estação Experimental de Campos dos Goytacazes–RJ; IAC: Instituto Agronômico de Campinas.

^3^ Analysis of resistance and susceptibility to brown rust corresponds to the analyses conducted in the field.

**Fig 1:**
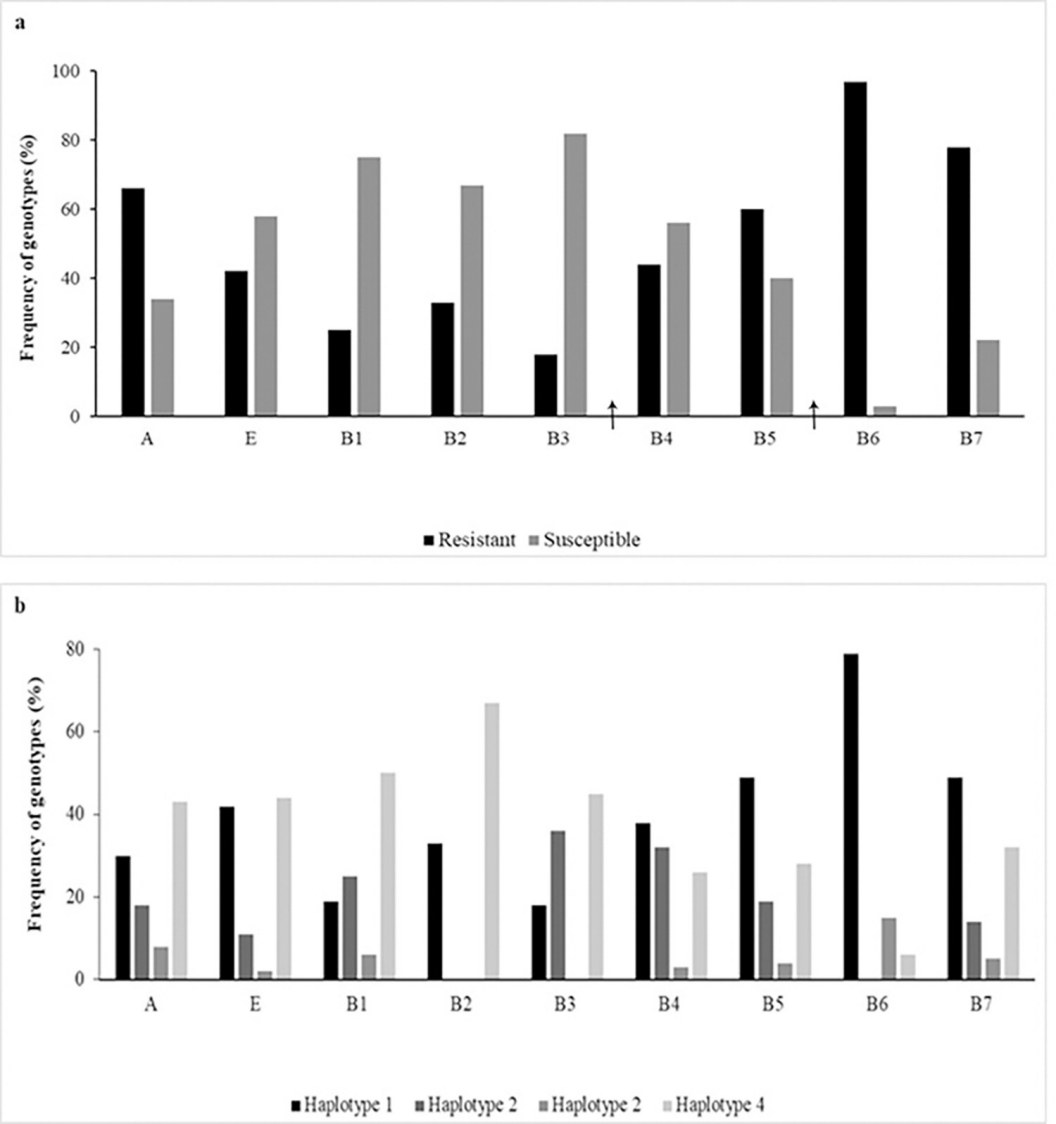
Field and molecular evaluation of brown rust response in sugarcane accessions. **a**. Frequency of genotypes resistant and susceptible to the disease according to a diagrammatic rating scale. Genotypes were categorized into groups: Ancestral germplasm (A), foreign (FH), and Brazilian (B), further subdivided into B1 (1940–1951), B2 (1952–1961), B3 (1962–1971), B4 (1972–1981), B5 (1982–1991), B6 (1992–2001), and B7 (2002–2011). Arrows indicate between B3 and B4, the first report of brown rust occurrence in commercial sugarcane areas worldwide (Comstock, [68]), and between B5 and B6, the emergence of the disease in Brazil (Copersucar 1986); **b.** Frequency of haplotypes of molecular markers linked to the *Bru1* gene in the A, FH, and B groups.

Out of a total of 188 accessions from Brazilian breeding programs (B), 116 (61.7%) were resistant to brown rust (Table 1 and S2A Fig). According to the breeding programs, resistance was detected in 94 RB accessions, 12 SP accessions (COPERSUCAR), one CTC accession (Centro de Tecnologia Canavieira), five IAC accessions, and four CB accessions (cultivars from Campos-RJ) (Table 1 and S2A Fig, and S1 Table). In the B1, B2, and B3 subgroups for the periods 1940–1951, 1952–1961, and 1961–1970, respectively, the number of resistant accessions was lower than that in subsequent periods (Fig 1A). From B4 (1972–1981) onwards, there was an increase in the number of genotypes resistant to brown rust, and a 32% increase in resistant accessions from B5 (1982–1991) to B6 (1992–2001) was observed (Fig 1A).

### Haplotype frequencies of molecular markers associated with the *Bru1* gene

Of the total number of evaluated accessions, 128 (42.6%) showed haplotype 1 (with both molecular markers linked to the *Bru1* gene), and 102 (34%) showed haplotype 4 (with both molecular markers linked to the *Bru1* gene missing). Only 17.3% of the accessions showed haplotype 2 (with only the R12H16 marker), and 6% showed haplotype 3 (with only the 9O20-F4-RsaI marker) (Tables 1 and S1).

The FH and B groups exhibited a greater frequency of haplotype 1 (82%) than did the ancestral germplasms (A) (18%). Among the improved Brazilian accessions (B), the highest proportion of the *Bru1* gene was detected in the RB genotypes (79%), followed by the CB (3.3%), IAC (4.4%), and SP (12.2%) genotypes (S1 Table and S2B Fig). In the ancestral germplasm (A), haplotype 1 was observed in representatives of the species *S*. *officinarum*, *S*. *sinense*, *S*. *barberi*, *S*. *robustum*, and *S*. *spontaneum*, with frequencies of 29.7%, 80%, 100%, 80%, and 14%, respectively (S1 Table).

On the other hand, the absence of markers for the *Bru1* gene was more frequent in the ancestral germplasms (A) (43.4%) than in the improved accessions (FH + B) (33.4%). For example, in group A, haplotype 4 was observed in 43% of *S*. *officinarum* accessions, six genotypes of *Erianthus* spp., and hybrids NG57-50 (*S*. *officinarum* x *S*. *spontaneum*) and IJ76-314 (*S*. *robustum* x S. officinarum). In the improved Brazilian germplasm (B), there were 53 accessions with haplotype 4 (S1 Table and S1B Fig). In the FH set, accessions from Argentina (NA56-79 and TUC71-7) and Australia (Q70, Q165, and Q117) had haplotype 4 (S1 Table).

Considering the Brazilian improved accessions (B) developed over seven decades (from 1940–1951 to 2002–2011), a 39% increase in the frequency of haplotype 1 was observed among accessions from the B1 and B7 sets (Fig 1B).

### Association between field response to brown rust and the *Bru1* gene

Haplotype 1 was found in 128 (70.7%) of the 181 resistant accessions in the field evaluations (Tables 1 and S1). In the improved Brazilian germplasms (B), a strong association between haplotype 1 and field resistance to the disease was also observed. Haplotype 1 was observed among 78.8% (71/90) of the RB accessions, 12.2% (11/90) of the SP accessions, 4.4% (4/90) of the IAC accessions, 3.5% (3/90) of the CB accessions, and 1.1% (1/90) of the CTC accessions. Foreign improved germplasm (FH) accessions that were resistant in the field evaluations also exclusively presented haplotype 1 (Tables 1 and S1).

In contrast, there was a 46% association between field resistance and haplotype 1 in the ancestral germplasm (A) accessions (Table 1). Out of the 38 representative *S*. *officinarum* accessions, 19 were field resistant, but only 11 were genotyped as haplotype 1 (Ajax, Black Borneo, Caiana Listrada, Cana Blanca, Ceram Red, IN84-105, IN84-106, NG57-221, Sabura, Sac. Offic. 8284, and White Transparent). Five *S*. *spontaneum* accessions were field resistant, but only Krakatau exhibited haplotype 1. Among the nine *S*. *robustum* accessions, only IM76-228 was field resistant and had haplotype 1. Among the five *S*. *sinense* accessions, four were field resistant, and all four displayed haplotype 1 (Agaul, Ar Chi, China, and Maneria). Finally, out of the four *S*. *barberi* accessions, three were field resistant and showed the presence of haplotype 1 (Chin, Chunnee, and Ganda Cheni) (S1 Table). In the ancestral germplasm (A), 16 accessions exhibited haplotype 4 and were field resistant, while 12 were susceptible. Among the foreign hybrids (FHs), all accessions with haplotype 4 were susceptible in the field. Among the 53 Brazilian hybrids with haplotype 4, only five were field resistant, and 48 were susceptible (Tables 1 and S1).

### Exploring potential sources of the *Bru1* gene in a Brazilian sugarcane cultivar

To trace the presence of the *Bru1* gene in modern sugarcane, the cultivar RB966928, which is planted over approximately 720 thousand hectares in the cultivated area of Brazil, was chosen. The genealogy of this cultivar is shown in S4 Fig, and it is composed of 67 sugarcane genotypes. Overall, thirty-three accessions were evaluated with the molecular markers R12H16 and 9O20-F4-RsaI. Of the 33 genotypes, 16 presented haplotype 1, indicating the presence of the *Bru1* gene; 15 presented haplotype 4, indicating the absence of the *Bru1* gene; and the remaining two accessions presented haplotype 3.

The results showed that haplotype 1, present in RB966928, was also found in ancestral genotypes such as Chunnee (*S*. *barberi*), White Transparent (*S*. *officinarum*), and Kassoer (hybrid between Black Cheribon (*S*. *officinarum*) and Glagah (*S*. *spontaneum*), as well as in improved accessions that were frequently used in crosses as part of Brazilian breeding programs, such as POJ2878. The cultivar R570, where the *Bru1* gene was originally identified, is not a part of the genealogy of RB966928. However, out of the total of 18 genotypes that make up the genealogy of R570 [43,44], eleven were also included in the genealogy of RB966928 (Chunnee, Black Cheribon, Glagah, Bandjarmasin Hitam, Loethers, Kassoer, EK28, POJ213, POJ100, POJ2364, and POJ2878). On the other hand, the accession POJ213 (hybrid between Chunnee (*S*. *barberi*) and Black Cheribon (*S*. *officinarum*), despite being resistant to brown rust, presented haplotype 4 (S5 Fig and S1 Table), suggesting an alternative source of disease resistance.

### Severity and incidence of orange rust in the field

The results of the phenotypic evaluation of the response to orange rust in the 300 accessions of the nuclear collection are summarized in Tables 2 and S1. A total of 289 accessions (96.3%) received ratings from 1 to 3 (resistant), and 11 accessions (3.7%) received ratings from 4 to 9 (susceptible), considering the average value over the two years of evaluation (S3A Fig).

**Table 2:** Field evaluation of orange rust reaction and genotyping of molecular markers for the *G1* gene in a nuclear collection of 299 sugarcane accessions.

	Resistant		Susceptible	
Access	Presence	Absence	Presence	Absence
**Ancestral access (A)**	27	48	1	0
**Foreign hybrids (FH)**	25	10	0	1
**Brazilian Breeding Programs (B)** ^**2**^				
**RB**	71	53	5	1
**SP**	11	16	0	3
**CTC**	1	1	0	0
**IAC**	10	3	0	0
**CB**	8	5	0	0
**Total**	152	136	6	5

^1^ *G1* marker to orange rust severity according to Yang et al., [31], indicates the presence (P) or absence (A).

^2^ Brazilian Breeding Programs: RB: ^2^ Brazilian Breeding Programs: RB: Rede Interuniversitária para o Desenvolvimento do Setor Sucroenergético; SP: Copersucar; CTC: Centro de Tecnologia Canavieira; CB: Estação Experimental de Campos dos Goytacazes–RJ; IAC: Instituto Agronômico de Campinas.

*Regarding the CP70-1547 genotype, there is no information available for the *G1* marker.

All accessions of *S*. *officinarum*, *S*. *spontaneum*, *Erianthus*, *S*. *sinense*, *S*. *barberi*, and *S*. *edule* exhibited resistance to orange rust (Table 2). Among the *S*. *robustum* accessions, approximately 85.7% showed resistance to orange rust, except for the genotype IJ76-318, which originated from Indonesia and showed susceptibility to the disease (S1 Table). Among the improved foreign accessions (FH), 34 (97.2%) accessions displayed resistance to orange rust (Table 2), including genotypes POJ2878 and R570, which originated from Indonesia and Reunion Island, respectively (S1 Table).

**Fig 2:**
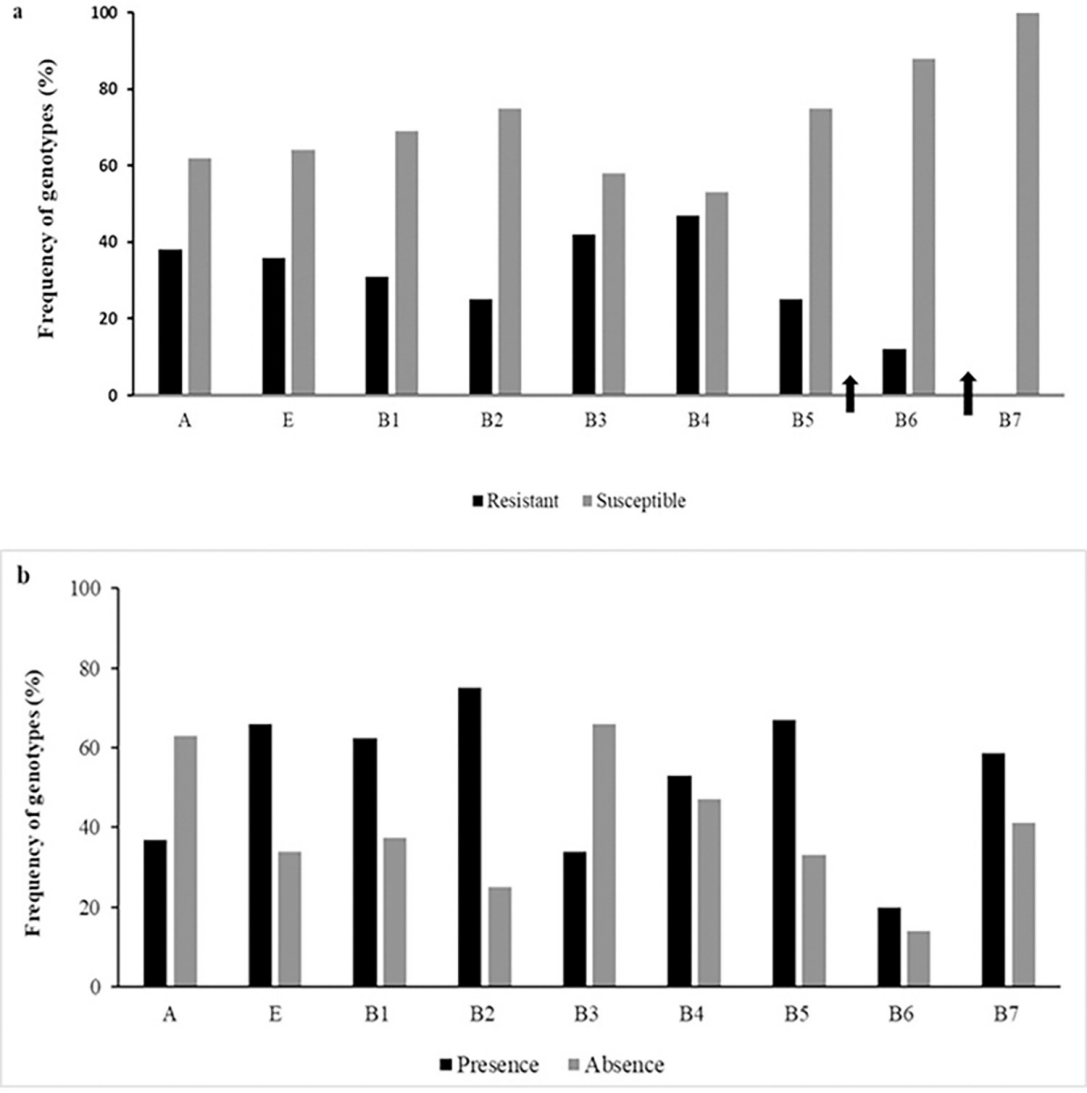
Frequency of accessions according to resistant and susceptible classes to orange rust and also according to the presence and absence of the molecular marker *G1*. **a.** Frequency of accessions in the resistant and susceptible classes to orange rust considering the ancestral germplasm (A), the set of improved foreign accessions (FH), and the set of improved Brazilian accessions (B), which was divided into subgroups according to the decade in which their parents were crossed; B1, B2, B3, B4, B5, B6, and B7, composed of accessions resulting from crosses made between the periods 1940–1951, 1952–1961, 1962–1971, 1972–1981, 1982–1991, 1992–2001, 2002–2011 respectively. The arrow in B6, indicates the first report of orange rust occurrence in commercial sugarcane areas in Australia, while the arrow B7 indicates the emergence of orange rust in Brazil in 2009 (Barbasso et al., [70]). **b.** Frequency of accessions according to the presence and absence of the molecular marker *G1* for the A, FH, and B subgroups.

Within the subset of improved Brazilian accessions (B), susceptibility was observed in B4 (1972–1981), B5 (1982–1991), and B6 (1992–2001). The presence of cultivars susceptible to the disease was not detected in B7 (2002–2011) and subsequent temporal groups (Fig 2A).

### Frequency of the *G1* molecular marker in the nuclear collection of accessions

Of the 299 accessions in the nuclear collection evaluated, 152 (52%) exhibited the presence of the *G1* marker (Table 2 and S3 Fig). The frequency of accessions with the *G1* marker was greater in the set of improved accessions (B + FH) (58%) than in the ancestral germplasm (A) (36.8%) (S3 Fig). Considering Brazilian breeding programs, there was variation in the presence of the *G1* marker, which was most frequently found in the IAC germplasm (75%). An examination of the subgroups of Brazilian accessions (B1 to B7) revealed that despite most cultivars being resistant to orange rust in the field (Fig 2A), the *G1* marker was not present in any of these cultivars (Fig 2B).

## Discussion

Fungal diseases that cause leaf rust pose significant challenges in sugarcane-producing countries worldwide [45,46]. Diseases such as brown and orange rust are associated with significantly reduced sugarcane crop yields and, consequently, critical economic losses [16,20,47]. The search for genetic resistance is an efficient and environmentally sustainable way to develop commercial sugarcane varieties [47,48]. However, the development of new rust-resistant cultivars is laborious, time-consuming, and expensive, as it involves resistance tests on natural infestations in the field for several years in different agricultural production environments [6,49]. Genetic tools, such as molecular markers, can aid in the molecular characterization of resistance alleles in the germplasm used by breeders and are helpful tools for discarding susceptible clones in the selection phase [6,50,51]. In our study, the field trials conducted showed that 60.3% of the accessions evaluated in a nuclear collection were resistant to brown rust under natural infection conditions.

The strong association between resistant accessions of improved Brazilian germplasms and haplotype 1 (Table 1) suggests that *Bru1* is the primary source of resistance to brown rust for Brazilian sugarcane genetic improvement programs. Here, we observed that 77.7% of the Brazilian germplasm accessions were field resistant and presented haplotype 1 for the *Bru1* gene. Considering the subsets B1 to B7 of improved Brazilian accessions, there was an increase in the number of genotypes resistant to brown rust over the decades, according to field evaluations. Costet et al. [30] evaluated 380 accessions from different countries in a field trial, 194 of which showed resistance to brown rust, and of these, 85.5% carried haplotype 1. Among the 94 main varieties from the Chinese sugarcane system, 70.21% were disease resistant, with the *Bru1* gene (haplotype 1) being present in 57.45% of the varieties. Dijoux et al. [16] evaluated 112 elite varieties belonging to four successive breeding series and reported that *Bru1* was present in 74.1% of the varieties but absent in 22.3% of the varieties and undetermined in the remaining 3.6%. In our study, all the foreign field-resistant hybrids also exhibited haplotype 1. Considering the ancestral germplasm, approximately 46% of the accessions were resistant to brown rust and carried haplotype 1 (Table 1). Taken together, these results indicate that *Bru1* was part of the gene pool of the ancestors of the *Saccharum* complex and was transmitted to modern allo-autopolyploid cultivars, overcoming the random pairing and recombination of chromosomes in meiosis through the selection and fixation of functional alleles [29,52,53]. Healey et al. [54] completed the full genome mapping of the sugarcane cultivar R570, covering the entire *Bru1* gene region. In this region, two candidate genes represent a tandem kinase-pseudo kinase (TKP) structure, TKP7 and TKP8, and were found as the causative genes for brown rust resistance. The suggested model is that these genes recognize fungal effectors and trigger a signaling cascade to confer resistance against the pathogen. The high frequency of the *Bru1* gene in Brazilian breeding programs suggests that this gene is the predominant source of resistance in Brazilian sugarcane cultivars, demonstrating the great effort of these programs in using resistant cultivars as parents for crossings [44]. This practice intensified mainly after the emergence of brown rust in Brazil in 1986, when Brazilian breeding programs began to prioritize the use of these resistant cultivars for such crossings. In particular, we observed an increase in the numbers of field-resistant accessions and accessions with *Bru1* (haplotype 1) in the data shown here (Fig 1A and 1B). In the present study, considering the improved Brazilian accessions (subgroups B1 to B7), there was an increase in the number of genotypes resistant to brown rust over the decades, according to phenotypic evaluations carried out in the field. This result suggests that breeding programs were efficient in fixing resistance alleles through crossing.

**Fig 3:**
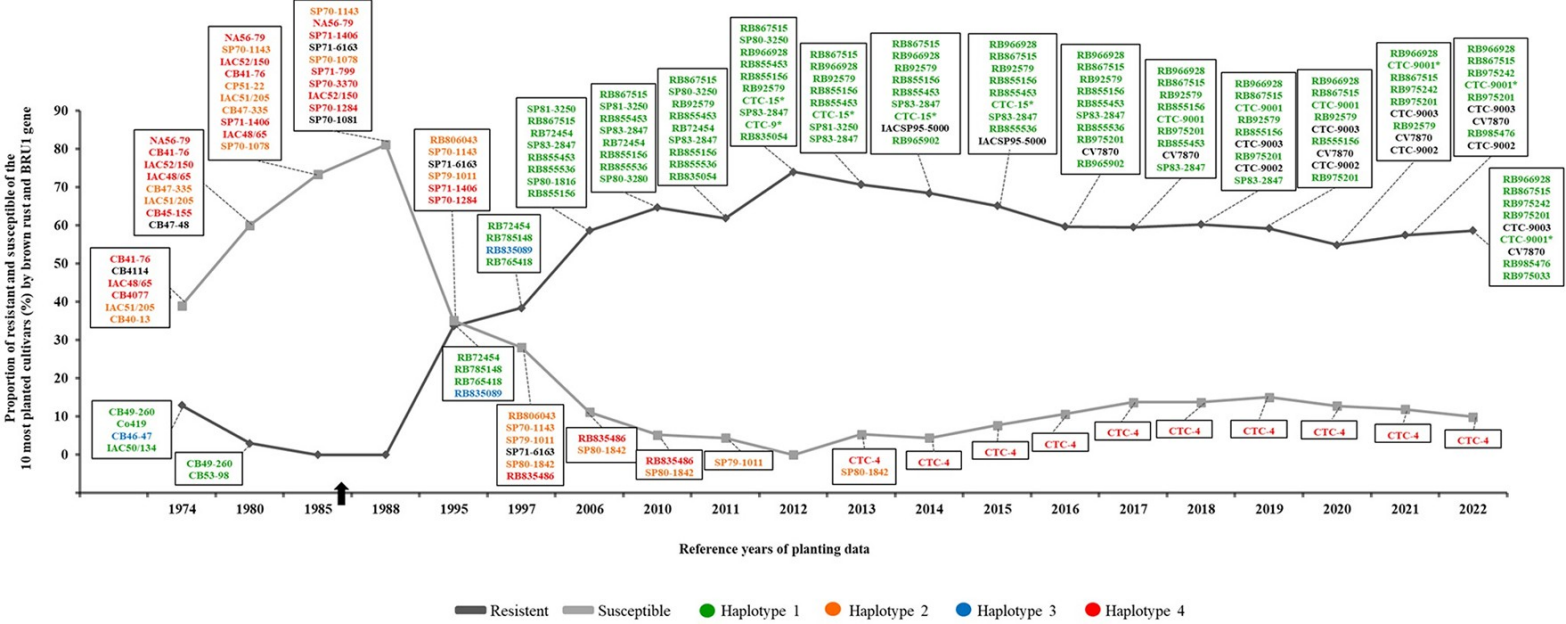
Timeline for the period between 1974 and 2022 depicting the proportion of resistant and susceptible cultivars among the top 10 most planted sugarcane cultivars in the states of São Paulo and Mato Grosso do Sul, in the Central-South region of Brazil. Cultivars CB41-14, CB47-48, SP71-6163, SP71-1081, IACSP95-5000, CV7870, CTC-9003, CTC-9002 lack available haplotype information for the *Bru1* gene. Black arrow: Indicates the year 1986 when brown rust emerged in Brazil.*Haplotype information sourced from Neuber et al., [55].

Although there may be alternative sources of resistance to brown rust, it appears that the *Bru1* gene has given the cultivars currently cultivated in Brazil durable genetic resistance. Some of the Brazilian commercial varieties resistant to brown rust have POJ2878 as a common ancestor. The presence of haplotype 1 in this ancestor suggests that it may have been one of those responsible for transmitting the *Bru1* gene. In general terms, genetic improvement programs focus on two main processes: a) crossings with the aim of generating genetic variability and b) selection phases (progeny assessment trials, clonal assessment trials and final assessment trials) [6]. Thus, the small number of genetic recombinations and the intense activity of genotype selection in different locations and years may have helped support the presence of the *Bru1* gene among commercial Brazilian cultivars. RB72454, the cultivar resistant to brown rust responsible for changing the cultivar scenario when the disease arrived in the country, as well as the ones most frequently planted in Brazil today (RB966928, RB867515, RB92579 and RB855156; see Fig 3 and S5 Fig), has haplotype 1 and the cultivar POJ2878 in its genealogy.

A survey of the ten most planted cultivars in the states of São Paulo (SP) and Mato Grosso do Sul (MS) (located in the southcentral region of Brazil) was conducted through a varietal census carried out between 1974 and 2022 [41]. In this set of genotypes, the reactions to brown rust and orange rust were assigned according to the diagrammatic scale in field evaluations. The presence of the *Bru1* gene and *G1* marker was also assessed for the same genotypes (Figs 3 and S4).

In 1974, six cultivars were susceptible to brown rust (CB41-76, CB41-14, IAC48/65, CB40-77, IAC51/205, and CB40-13), accounting for almost 40% of the sugarcane-planted area in the southcentral region, an extent of 480 thousand hectares. By 1980, eight cultivars were susceptible to brown rust (NA56-79, CB41-76, IAC52/150, IAC48/65, CB47-355, IAC51/205, CB45-155, and CB47-48), covering approximately 60% of the area (approximately 660 thousand hectares) (Fig 3). In 1986–88, brown rust was detected for the first time in Brazil, and commercial sugarcane fields were dominated by susceptible cultivars. Most susceptible cultivars exhibited haplotypes 2 and 4 for the *Bru1* gene, such as the most planted SP70-1143, NA56-79, and SP71-1406. In 1986, brown rust was detected for the first time in Brazil. Resistant cultivars, although less planted between 1974 and 1988, presented haplotype 1 (CB49-260, Co419, IAC50/134, and CB53-98), indicating the presence of the *Bru1* gene, and haplotype 3 (CB46-47) (Fig 3). Eleven years after the arrival of brown rust in Brazil, the resistant cultivars RB72454, RB785148, RB835089, and RB765418 occupied approximately 1 million hectares, compared with the 700 thousand hectares cultivated with susceptible genotypes. These resistant cultivars exhibited haplotype 1, except for RB835089 (haplotype 3).

With the expansion of sugarcane cultivation in Brazil, resistant cultivars harbouring the *Bru1* gene began to occupy increasingly larger areas. In 2012, the ten most planted cultivars showed field resistance and haplotype 1, occupying approximately 3.6 million hectares. In the following years, between 2013 and 2015, susceptible cultivars (CTC-4 and SP80-1842) were among the most planted, but the combined area of resistant cultivars with haplotype 1 was greater, at approximately 3.8 million hectares. In the last six years (2016 to 2022), the most planted cultivar was RB966928, which is resistant to the disease and contains the *Bru1* gene (Fig 3).

From the first to the more recent survey, a period from 1974 to 2022, the sugarcane area in the Centro-Sul region (states of SP and MS) increased from approximately 1.2 to 6.1 million hectares, a fivefold expansion. Conversely, considering the area occupied by the 10 most planted varieties, there was an increase in the frequency of the *Bru1* gene due to the predominant cultivation of resistant cultivars with haplotype 1.

Overall, the data indicated the prevalence of cultivars with field resistance to orange rust during the period from 1974 to 2022. In 1995 and 1997, the RB72454 cultivar was among the most planted, accounting for approximately 24% of the planted area. However, this cultivar showed susceptibility in the field when exposed to the pathogen, despite possessing the *G1* marker. From 2006 to 2013, the SP81-3250 cultivar was among the most planted, accounting for approximately 12% of the planted area. This cultivar, in turn, showed susceptibility to orange rust and lacked the *G1* marker. Others were swiftly planted to replace it, and it ceased to be among the most planted after 2013. From 2016 onwards, no orange rust-susceptible cultivar was included among the top 10 most planted cultivars.

Interestingly, the CTC-4, SP83-2847, RB975201, RB975242, RB985476, and RB975033 cultivars are field resistant but do not exhibit the *G1* marker. This finding suggests the existence of an alternative source of resistance to orange rust in these cultivars (S4 Fig). In the breeding programs of Louisiana (United States) and Obispo Colombres (Argentina) [21,56], the frequency of *Bru1* is low, at 6% and 7%, respectively. In Argentina, climatic conditions favour a high level of pathogen variability [57,58] and, consequently, increase the possibility of the emergence of virulent genotypes that overcome resistance from *Bru1*. These results suggest the predominance of an alternative source of resistance to brown rust or the occurrence of races of *P*. *melanocephala* that *Bru1* does not confer resistance to [28,59,60]. Studies are being conducted to develop molecular markers for alternative resistance sources [27,44], to improve prediction of the *Bru1* marker through the use of new restriction enzymes [61,62], and even to include the *Bru1* gene as a fixed effect in genomic prediction models, which could enhance prediction accuracy [63]. Distortions in the pattern of joint segregation of molecular markers, represented by haplotypes 2 and 3, may highlight rare recombination effects [30], non-complementarity of primer sequences in the evaluated accessions [26] or yet another source of resistance to brown rust. This alternative source of resistance has great potential for identification in the field, as resistant accessions do not present either of the two markers for the *Bru1* gene [28,30,59,64,65], represented by haplotype 4. Considering the ancestral germplasm, all species evaluated had at least one phenotypically resistant representative with haplotype 4, except for *S*. *barberi*, which had four with haplotype 1 and one with haplotype 2 (Tables 1 and S1). Although the *Bru1* gene is a main responsible for resistance to brown rust, it is important that controlled experiments be carried out, especially in regions with climatic conditions favourable for the occurrence of the pathogen, to identify alternative resistance genes, select resistant clones that do not harbour the *Bru1* gene, include these clones in breeding efforts to broaden the genetic basis of resistance to brown rust [26,28,51,56,62] and minimize the risks of the resistance gene losing its efficacy [19,66,67]. Another interesting point is that cultivars that have some level of susceptibility to brown rust are still cultivated, thus reducing the selection pressure for a possible more virulent race of the pathogen and the consequent breakdown of resistance provided by *Bru1*. So, these areas with susceptible cultivars, even on a smaller scale, serve as refuge areas.

Orange rust in sugarcane is a disease with a more recent occurrence in commercial fields than brown rust. However, the effect of the fungus *P*. *kuehnii* was devastating in the 2000s in Australia, with considerable economic losses and production losses of Aus$150–210 million [68]. This disease was first reported in Florida in 2007 [69]. It was next detected in Asia, Oceania, and Central and North America, and at the end of 2009, orange rust was reported for the first time in Brazil [70]. Yang et al. [31] developed a molecular marker associated with an orange rust resistance gene, called *G1*. This marker predicted 65.8% of resistant phenotypes in a mapping population of 165 sugarcane genotypes.

In the present study, most accessions (96.3%) were resistant to the fungus *P*. *kuehnii* when evaluated under natural inoculation conditions. Of the 288 resistant accessions from the nuclear collection, 52.7% had the *G1* marker. These data indicate that although this disease is recent in Brazil, some resistant cultivars were already being planted before the arrival of the pathogen. This was important for preventing large productivity losses due to orange rust in Brazilian cultivation areas, allowing the rapid replacement of susceptible cultivars [32]. A similar study of 3375 accessions of *Saccharum* spp. cultivars and allied genera belonging to the ICAR-Sugarcane Breeding Institute (Research Centre, Kannur) revealed that approximately 98% of the clones were resistant to orange rust [71].

Hoepers et al. [72] evaluated 80 sugarcane genotypes for the presence of the *G1* marker and concluded that the marker had low efficiency for predicting resistance to orange rust in clones selected in a genetic improvement program. On the other hand, Fier et al. [32] observed high efficiency in predicting resistance using the *G1* marker (71.43%) when evaluating 24 Brazilian commercial cultivars. Borella et al. [73] evaluated 63 sugarcane progenitors preserved in the RIDESA germplasm bank and found that the molecular marker *G1* was 71% accurate in predicting the resistance phenotype and thus could be used to characterize germplasm. There are different ways to classify resistant genotypes using a rating scale, and there is some evidence that more aggressive isolates or races of *P*. *kuehnii* [74–76] influence the predictive efficiency of the *G1* marker.

In our study, the frequency of the *G1* marker in the ancestors was lower than that in the foreign hybrids and Brazilian accessions. Predominance of the *G1* marker was also observed in Brazilian accessions (B1-B7), even when no resistance response to the pathogen was observed in field evaluation. Yang et al. [31] proposed that the resistance gene associated with the *G1* marker is responsible for horizontal resistance (durable resistance). Alternatively, the search for and development of other molecular markers in greater linkage disequilibrium with the gene linked to *G1* could result in greater efficiency in predicting resistance to orange rust. Using an association mapping strategy, McCord et al. [77] detected 10 marker SNPs that were statistically significant for the quantitative measurement of orange rust severity.

In the study conducted by Dijoux et al. [78], a detailed assessment was carried out on 568 modern interspecific hybrids of sugarcane (*Saccharum officinarum* x *S*. *spontaneum*) from the Réunion breeding program, focusing on resistance to orange rust under natural infection conditions. Through a single-locus genome-wide association study (SL-GWAS), five quantitative trait loci (QTLs) were identified, all originating from *S*. *spontaneum*. Additionally, a multi-locus GWAS (ML-GWAS) identified an additional, albeit less significant, resistance QTL originating from *S*. *officinarum*. The analysis revealed that all six QTLs exhibited a moderate to significant phenotypic effect on orange rust resistance.

The polygenic inheritance of resistance to orange rust hampers to develop tools capable of early identification of resistant clones. In this context, the G1 is an initial source of research about the defence mechanisms to orange rust. The association between phenotypic and genotypic data should also be better evaluated, since genotypes with some level of infection, up to 3 on a diagrammatic scale, could be considered resistant. Although immunity hardly exists, when a pathogen quickly spreads, it is important to identify genotypes that tolerate the disease and gradually increase selection pressure to obtain plants that have efficient defence mechanisms without compromising their productivity. In Brazil, even before the arrival of the disease, breeding programs exchanged some important cultivars with countries where orange rust was already present to discover resistance. This process would be faster and more efficient if molecular tools with predictive capacity were quickly developed and made available to the scientific community. One of the main bottlenecks in accelerating the development of sugarcane cultivars with desirable characteristics, such as disease resistance, is the lack of a large-scale methodology that can be carried out quickly and accurately with little time demand. The use of high-throughput phenotyping methods for brown and orange rust through multispectral data based on UAV and machine learning algorithms could be an innovative alternative in the search for resistant genotypes in sugarcane breeding programs [79,80]. The contribution of these technologies is important in the context of the field selection phases, mainly in the early phases where the selection pressure is to discard susceptible genotypes regardless of the type of rust, since there is a tendency to confuse brown and orange rust in the images collected. Collaboration to develop more efficient and faster selection strategies is essential to enable the early detection of resistant cultivars. On the other hand, when the objective is to increase the frequency of rust resistance alleles in breeding programs, characterize germplasm banks, or screen genotypes used in germplasm exchanges, the molecular markers 9020-F4-RsaI, R12H16 (*Bru1*) and *G1* are potential tools for the diagnosis and prediction of resistant phenotypes. There is a clear need to search for new molecular markers based on genetic mapping strategies that aim to increase linkage disequilibrium in regions previously associated with resistance, also considering alternative sources of resistance and that account for the genetic diversity of the pathogens *P*. *melanocephala* and *P*. *kuehnii*.

## Supporting information

S1 Table Sugarcane nuclear collection evaluated in this study. Total of 300 sugarcane accessions evaluated in the field for response to resistance (R) or susceptibility (S) to brown and orange rust diseases, haplotypes of the *Bru*1 based on the R12H16 and 9O20-F4-RsaI markers, and presence (1) or absence (0) of the *G1* marker. The phenotypic average of brown and orange rust obtained through field trials were also presented. BP: accessions from breeding program; NA: information not available.
(PDF)

S1 Fig Different levels of severity of *Puccinia melanocephala* (brown rust) an*d Puccinia kuehnii* (orange rust) on sugarcane leaves. In the phenotypic evaluation, a diagrammatic scale was used with scores for disease severity (Amorim et al., [37]); **S1A Fig.** Leaf without disease symptom for brown rust; **S1B Fig.** Leaf with grade 4 on the diagrammatic scale for brown rust; **S1C Fig.** Leaf with a score of 6 on the diagrammatic scale for brown rust; **S1D Fig.** Leaf without the disease symptom for orange rust; **S1E Fig.** Leaf with a grade of 8 on the diagrammatic scale for orange rust.
(PDF)

S2 Fig Frequency of accessions in response to brown rust and the *Bru1* gene. **S2A Fig.** Phenotypic frequency of resistant and susceptible accessions to brown rust; and **S2B Fig.** Frequency of *Bru1* haplotypes in the nuclear collection of the 300 accessions.
(PDF)

S3 Fig Frequency of accessions in response to orange rust and the molecular marker *G1*. **S3A Fig.** Phenotypic frequency of accessions resistant and susceptible to orange rust; **S3B Fig.** Genotypic frequency of the presence or absence of the *G1* marker in the nuclear collection of the 300 accessions. *Regarding the CP70-1547 genotype, there is no information available for the *G1* marker.
(PDF)

S4 Fig Proportion of resistant and susceptible cultivars among the 10 most planted sugarcane cultivars in the states of São Paulo and Mato Grosso do Sul, Central-South region of Brazil, before and after the emergence of orange rust in Brazil. Cultivars in black do not have available information for the *G1* molecular marker.
(PDF)

S5 Fig Genealogy of the cultivar RB966928. Accessions indicated with filled triangles, circles, and squares in black represent haplotypes 1, 3, and 4, respectively, for molecular markers associated with the *Bru1* gene. The kinship matrix was provided by the Sugarcane Breeding Program at the Federal University of São Carlos, part of the Interuniversity Network for the Development of the Sugarcane Sector (RIDESA) (https://www.ridesaufscar.com.br/). PedigraphTM software was used to construct the genealogical tree. In the image, (R): accessions resistant to brown rust; (S): accessions susceptible to brown rust. * Information collected from the references: Costet et al., [30], Glynn et al., [56], Racedo et al., [59], Parco et al., [26], and Neuber et al., [55].
(PDF)
