# Supplementary material for: Molecular evaluation and phenotypic screening of brown and orange rust in Saccharum germplasm
Source: PLoS One. 2024 Jul 30;19(7):e0307935. doi: 10.1371/journal.pone.0307935 (PMC11288420; doi:10.1371/journal.pone.0307935)
Supplement: S1 Table — Total of 300 sugarcane accessions evaluated in the field for response to resistance (R) or susceptibility (S) to brown and orange rust diseases, haplotypes of the Bru1 based on the R12H16 and 9O20-F4-RsaI markers, and presence (1) or absence (0) of the G1 marker. The phenotypic average of brown and orange rust obtained through field trials were also presented. BP: accessions from breeding program; NA: information not available. (PDF) [file pone.0307935.s001.pdf]

Accessions	Representative genotype of	Country	Groups	*Bru1* haplotype	Brown rust phenotype	Phenotypic average of brown rust	*G1* marker	Orange rust phenotype	Phenotypic average of orange rust
28NG289	*S. robustum*	NA	A	4	R	1,0	0	R	1,0
57NG12	*S. robustum*	NA	A	4	R	1,0	0	R	1,0
75//09 ERIANTHUS	*Erianthus* spp.	NA	A	4	R	1,0	1	R	1,0
AGAUL	*S. sinense*	África do Sul[b]	A	1	R	1,0	0	R	1,0
AGOULE	*S. barberi* x ?	NA	A	1	R	1,0	1	R	1,0
AJAX	*S. officinarum*	NA	A	1	R	1,0	1	R	1,8
AKBAR	BP	NA	E	4	S	2,3	0	R	1,0
AR CHI	*S. sinense*	Taiwan[b]	A	1	R	1,0	0	R	1,0
ARUNDOID B	BP	USA[b]	E	4	S	1,2	1	R	1,0
BADILA	*S. officinarum x* NG9615	Nova Guiné[a]	A	2	R	1,0	0	R	1,2
BLACK BORNEO	*S. officinarum*	índonesia[b]	A	1	R	1,0	0	R	2,0
CAIANA FITA	*S. officinarum*	NA	A	4	S	1,3	0	R	1,5
CAIANA LISTRADA	*S. officinarum*	NA	A	1	R	1,0	0	R	1,3
CAIANA RISCADA	*S. officinarum*	NA	A	4	S	1,8	1	R	1,4
CAIANA VERDADEIRA	*S. officinarum*	NA	A	2	S	1,2	0	R	1,0
CANA ALHO	*S.officinarum*	NA	A	4	S	1,4	0	R	1,5
CANA BLANCA	*S. officinarum*	NA	A	1	R	1,0	1	R	1,7
CANA MANTEIGA	*S. officinarum*	NA	A	4	S	1,1	1	R	1,6
CB36-14	BP	Brasil[a]	B1	4	S	6,2	0	R	1,0
CB36-24	BP	Brasil[a]	B1	2	S	3,8	0	R	1,4
CB36-25	BP	Brasil[a]	B1	4	S	4,9	1	R	1,0
CB36-68	BP	Brasil[a]	B1	1	R	1,0	0	R	1,6
CB40-13	BP	Brasil[a]	B1	2	S	2,9	1	R	2,0
CB40-77	BP	Brasil[a]	B1	4	S	1,4	1	R	2,2
CB41-76	BP	Brasil[a]	B1	4	S	2,1	1	R	1,9
CB45-155	BP	Brasil[a]	B1	4	S	3,3	1	R	1,0
CB45-3	BP	Brasil[a]	B1	4	S	2,9	0	R	1,0

CB46-47	BP	Brasil[a]	B1	3	R	1,0	1	R	1,6
CB47-355	BP	Brasil[a]	B1	2	S	3,7	0	R	1,0
CB49-260	BP	Brasil[a]	B1	1	R	1,0	1	R	1,5
CB53-98	BP	Brasil[a]	B2	1	R	1,0	1	R	1,4
CERAM RED	*S. officinarum*	NA	A	1	R	1,0	0	R	1,0
CHIN	*S. barberi*	índia[a]	A	1	R	1,0	0	R	1,0
CHINA	*S. sinense*	NA	A	1	R	1,0	0	R	1,0
CHUNNEE	*S. barberi*	índia[b]	A	1	R	1,0	0	R	1,0
CIMCA77-316	BP	Bolívia[c]	E	2	S	5,8	1	R	1,0
Co290	BP	índia[c]	E	4	S	2,9	1	R	1,0
Co331	BP	índia[c]	E	2	S	4,2	1	R	1,0
Co419	BP	índia[c]	E	1	R	1,0	1	R	1,8
Co449	BP	índia[c]	E	4	S	4,1	1	R	1,0
Co740	BP	índia[c]	E	1	R	1,0	1	R	1,1
Co997	BP	índia[c]	E	1	R	1,0	1	R	2,2
CP51-22	BP	USA[c]	E	2	S	4,7	1	R	1,0
CP52-68	BP	USA[c]	E	1	R	1,0	0	R	1,5
CP53-76	BP	USA[c]	E	1	R	1,0	0	R	1,0
CP70-1547	BP	USA[c]	E	1	R	1,0	0	R	2,7
CR72/106	BP	R. Dominicana[c]	E	2	S	3,6	0	R	3,0
CRIOLLA MORADA	*S. officinarum*	NA	A	4	S	1,6	0	R	2,1
CRIOLLA RAYADA	*S. officinarum*	NA	A	4	R	1,0	0	R	1,0
CRISTALINA	*S. officinarum*	NA	A	4	R	1,0	0	R	1,7
D11/35	*S. officinarum*	USA[c]	E	4	S	2,1	0	R	1,6
D152	BP	NA	E	1	R	1,0	1	R	1,3
D625	BP	NA	E	4	S	3,2	1	R	1,7
F150	BP	Taiwan[c]	E	1	R	1,0	1	R	2,0
F31-962	BP	Taiwan[a]	E	1	R	1,0	1	R	1,6
F36-819	BP	Taiwan[a]	E	1	R	1,0	0	S	4,1

F76-1762	*Miscanthus x Erianthus* spp.	NA	A	4	S	1,4	1	R	1,2
FORMOSA	*S. officinarum*	Taiwan[c]	A	2	S	1,9	0	R	1,0
GANDA CHENI	*S. barberi*	índia[b]	A	1	R	1,0	0	R	1,0
H. KAWANDANG	*Erianthus* spp. x ?	Indonésia	A	4	R	1,0	1	R	1,0
H53-3989	BP	Estados Unidos[c]	E	4	S	3,0	0	R	1,0
H59-1966	BP	EstadosUnidos	E	1	R	1,0	0	R	2,9
HJ5741	BP	Jamaica[c]	E	1	R	1,0	0	R	2,4
IAC48-65	BP	Brasil[a]	B1	4	S	2,6	0	R	1,0
IAC49-131	BP	Brasil[a]	B1	4	S	2,4	1	R	1,3
IAC50-134	BP	Brasil[a]	B1	1	R	1,0	1	R	2,7
IAC51-205	BP	Brasil[a]	B1	2	S	3,8	1	R	1,0
IAC52-150	BP	Brasil[a]	B2	4	S	3,3	0	R	1,0
IAC58-480	BP	Brasil[a]	B2	4	S	3,6	1	R	1,8
IAC64-257	BP	Brasil[a]	B3	1	R	1,0	1	R	1,5
IAC68-12	BP	Brasil[a]	B3	1	R	1,0	1	R	1,6
IAC82-2045	BP	Brasil[a]	B5	3	R	1,0	1	R	1,7
IAC82-3092	BP	Brasil[a]	B5	4	S	1,4	1	R	1,0
IAC83-4157	BP	Brasil[a]	B5	2	S	2,6	1	R	1,0
IAC86-2210	BP	Brasil[a]	B5	1	R	1,0	0	R	1,1
IAC87-3396	BP	Brasil[a]	B5	2	S	1,8	1	R	1,1
IJ76-293	*S. robustum*	Indonésia[b]	A	3	S	1,3	0	R	1,0
IJ76-313	*S. officinarum*	Indonésia[b]	A	4	S	1,5	0	R	1,1
IJ76-314	*S. robustum x S. officinarum*	Indonésia[b]	A	4	S	1,1	0	R	1,7
IJ76-317	*S. officinarum*	Indonésia[b]	A	4	S	1,3	0	R	1,2
IJ76-318	*S. robustum* x ?	Indonésia[b]	A	2	R	1,0	1	S	3,9
IJ76-325	*S. officinarum*	Indonésia[b]	A	2	R	1,0	0	R	1,0
IJ76-326	*S. officinarum* x ?	Indonésia[b]	A	4	R	1,0	0	R	1,0
IJ76-360	*S. edule*	Indonésia[b]	A	4	R	1,0	1	R	1,0
IJ76-418 RED	*S. officinarum*	Indonésia[b]	A	2	R	1,0	0	R	1,0

IM76-227	*Erianthus* spp.	Indonésia[b]	A	4	R	1,0	0	R	1,0
IM76-228	*S. robustum*	Indonésia[b]	A	1	R	1,0	0	R	1,0
IM76-229	*S. robustum*	Indonésia[b]	A	3	S	1,1	0	R	1,0
IN84-103	*S. officinarum*	Indonésia[b]	A	2	S	2,1	0	R	1,0
IN84-104	*S. robustum* x ?	Indonésia[b]	A	3	R	1,0	0	R	1,0
IN84-105	*S. officinarum*	Indonésia[b]	A	1	R	1,0	1	R	1,0
IN84-106	*S. officinarum*	Indonésia[b]	A	1	R	1,0	0	R	1,3
IN84-117	*S. robustum*	Indonesia[b]	A	3	S	1,0	0	R	1,0
IN84-46	*S. officinarum*	Indonésia[b]	A	4	S	2,9	0	R	1,0
IN84-58	*S. spontaneum*	Indonésia[b]	A	2	R	1,0	1	R	1,0
IN84-73	*Erianthus* spp.	NA	A	4	R	1,0	0	R	1,0
IN84-77	*Erianthus spp.*	NA	A	4	R	1,0	0	R	1,0
IN84-82	*S. spontaneum*	Indonésia[b]	A	2	R	1,0	1	R	1,0
IN84-83	*Erianthus* spp.	NA	A	4	R	1,0	0	R	1,0
IN84-88	*S. spontaneum*	Indonésia[b]	A	4	R	1,0	1	R	1,1
IS76-155	*S. officinarum*	NA	A	2	R	1,0	1	R	1,6
KRAKATAU	*S. spontaneum*	NA	A	1	R	1,0	1	R	1,0
L60-14	BP	Estados Unidos[c]	E	4	S	1,3	1	R	1,0
LAUKONA	*S. officinarum* x ?	Estados Unidos[b]	A	1	R	1,0	0	R	1,7
LOUSER	*S. officinarum* x ?	NA	A	4	S	1,1	0	R	1,3
MALI	BP	NA	E	3	S	2,2	1	R	2,4
MANA II	BP	NA	E	4	S	1,4	1	R	1,0
MANERIA	*S. sinense*	Índia[b]	A	1	R	1,0	0	R	1,0
MUNTOK JAVA	*S. officinarum*	Indonésia[b]	A	2	R	1,0	1	R	1,6
MZ-151	*S. officinarum*	NA	A	2	S	1,2	0	R	2,1
NA56-79	BP	Argentina[a]	E	4	S	2,8	1	R	1,0
NCo 310	BP	África do sul[a]	E	1	R	1,0	1	R	1,0
NG21-17	*S. officinarum*	Nova Guiné[b]	A	4	S	1,1	0	R	1,1
NG21-21	*S. officinarum*	Nova Guiné[b]	A	4	S	3,1	1	R	1,0
NG57-221	*S. officinarum*	Nova Guiné[b]	A	1	R	1,0	1	R	1,1

NG57-50	*S.officinarum x S.spontaneum*	Nova Guiné[c]	A	4	S	3,4	1	R	1,1
NG77-18	*S. officinarum*	Nova Guiné[b]	A	4	S	4,0	0	R	1,0
POJ161	BP	Java[c]	E	4	S	4,0	1	R	1,3
POJ2878	BP	Java[c]	E	1	R	1,0	1	R	1,7
Q117	BP	Australia[a]	E	4	S	4,0	1	R	1,8
Q165	BP	Australia[a]	E	4	S	4,0	1	R	1,9
Q70	BP	Australia[a]	E	4	S	4,0	1	R	1,5
R570	BP	Ilhas Reunião[a]	E	1	R	1,0	0	R	1,8
RB002601	BP	Brasil[a]	B6	1	R	1,0	1	R	1,4
RB002700	BP	Brasil[a]	B6	1	R	1,0	1	R	1,6
RB002754	BP	Brasil[a]	B6	1	R	1,0	1	R	1,0
RB721012	BP	Brasil[a]	B4	4	S	4,8	1	R	1,0
RB72199	BP	Brasil[a]	B4	3	R	1,0	1	R	1,4
RB72454	BP	Brasil[a]	B4	1	R	1,0	1	S	3,5
RB725053	BP	Brasil[a]	B4	4	S	3,9	0	R	2,2
RB725828	BP	Brasil[a]	B4	2	S	4,8	0	R	1,0
RB732577	BP	Brasil[a]	B4	4	S	4,1	0	R	1,0
RB735200	BP	Brasil[a]	B4	1	R	1,0	0	R	1,0
RB735220	BP	Brasil[a]	B4	2	S	2,8	1	R	1,0
RB735275	BP	Brasil[a]	B4	4	S	5,1	0	R	1,1
RB736018	BP	Brasil[a]	B4	4	S	2,5	1	R	1,0
RB739735	BP	Brasil[a]	B4	1	R	1,0	1	R	1,4
RB75126	BP	Brasil[a]	B4	1	R	1,0	1	R	2,9
RB765418	BP	Brasil[a]	B4	1	R	1,0	1	R	2,3
RB785148	BP	Brasil[a]	B4	1	R	1,0	0	R	2,0
RB785750	BP	Brasil[a]	B4	2	R	1,0	1	R	2,3
RB806043	BP	Brasil[a]	B4	2	S	1,0	0	R	2,6
RB815521	BP	Brasil[a]	B4	4	S	4,5	1	R	1,6
RB815627	BP	Brasil[a]	B4	2	S	2,7	1	R	1,1

RB815690	BP	Brasil[a]	B4	4	S	2,6	1	R	1,0
RB825317	BP	Brasil[a]	B5	4	S	2,3	1	R	1,0
RB825336	BP	Brasil[a]	B5	2	S	1,3	0	R	1,0
RB825548	BP	Brasil[a]	B5	1	R	1,0	1	R	1,0
RB83100	BP	Brasil[a]	B5	4	S	3,5	0	R	1,0
RB83102	BP	Brasil[a]	B5	4	S	4,4	0	R	1,0
RB83160	BP	Brasil[a]	B5	4	S	2,5	1	R	1,0
RB835019	BP	Brasil[a]	B5	1	R	1,0	1	R	1,0
RB835054	BP	Brasil[a]	B5	1	R	1,0	1	R	1,1
RB835089	BP	Brasil[a]	B5	3	R	1,0	1	R	1,1
RB835205	BP	Brasil[a]	B5	1	R	1,0	1	R	1,3
RB835486	BP	Brasil[a]	B5	4	S	3,4	0	R	1,6
RB975242	*BP*	Brasil[a]	B6	1	R	1,0	0	R	1,3
RB845197	BP	Brasil[a]	B5	1	R	1,0	1	R	2,3
RB845210	BP	Brasil[a]	B5	1	R	1,0	0	R	1,4
RB845239	BP	Brasil[a]	B5	2	S	1,1	1	R	2,0
RB845257	BP	Brasil[a]	B5	1	R	1,0	1	R	1,2
RB845286	BP	Brasil[a]	B5	4	S	2,4	0	R	1,8
RB855002	BP	Brasil[a]	B5	1	R	1,0	1	R	2,9
RB855035	BP	Brasil[a]	B5	4	S	2,2	1	R	1,9
RB855036	BP	Brasil[a]	B5	1	R	1,0	0	S	3,9
RB855063	BP	Brasil[a]	B5	2	R	1,0	1	R	1,0
RB855070	BP	Brasil[a]	B5	4	S	1,1	1	R	1,0
RB855077	BP	Brasil[a]	B5	4	S	1,5	1	R	2,2
RB855113	BP	Brasil[a]	B5	1	R	1,0	0	R	1,0
RB855156	BP	Brasil[a]	B5	1	R	1,0	1	R	1,6
RB855196	BP	Brasil[a]	B5	1	R	1,0	1	S	3,0
RB855206	BP	Brasil[a]	B5	1	R	1,0	1	R	1,8
RB855350	BP	Brasil[a]	B5	1	R	1,0	0	R	1,3
RB855357	BP	Brasil[a]	B5	1	R	1,0	1	R	2,5

RB855453	BP	Brasil[a]	B5	1	R	1,0	1	R	1,4
RB855463	BP	Brasil[a]	B5	4	S	2,2	1	R	1,8
RB855465	BP	Brasil[a]	B5	2	R	1,0	0	R	1,0
RB855511	BP	Brasil[a]	B5	1	R	1,0	1	S	3,2
RB855533	BP	Brasil[a]	B5	1	R	1,0	1	R	2,4
RB855536	BP	Brasil[a]	B5	1	R	1,0	1	R	1,3
RB855546	BP	Brasil[a]	B5	1	R	1,0	0	R	2,5
RB855563	BP	Brasil[a]	B5	2	S	3,1	1	R	1,5
RB855574	BP	Brasil[a]	B5	4	S	2,8	1	R	1,0
RB855589	BP	Brasil[a]	B5	2	S	2,1	0	R	1,2
RB855595	BP	Brasil[a]	B5	2	R	1,0	1	R	1,0
RB855598	BP	Brasil[a]	B5	4	S	1,7	0	R	1,0
RB865214	BP	Brasil[a]	B5	4	S	3,0	0	R	1,0
RB867515	BP	Brasil[a]	B5	1	R	1,0	1	R	1,2
RB925211	BP	Brasil[a]	B6	1	R	1,0	1	S	3,5
RB925268	BP	Brasil[a]	B6	1	R	1,0	1	R	1,1
RB925345	BP	Brasil[a]	B6	4	S	3,1	1	R	1,0
RB92579	BP	Brasil[a]	B6	1	R	1,0	1	R	1,6
RB935744	BP	Brasil[a]	B6	1	R	1,0	1	R	2,4
RB965902	BP	Brasil[a]	B6	1	R	1,0	1	R	1,0
RB965917	BP	Brasil[a]	B6	1	R	1,0	1	R	1,1
RB966928	BP	Brasil[a]	B6	1	R	1,0	1	R	1,0
RB975148	BP	Brasil[a]	B6	1	R	1,0	1	S	3,5
SABURA	*S. officinarum*	NA	A	1	R	1,0	0	R	1,0
SAC.OFFIC.8272	*S. officinarum*	Malasia[b]	A	2	S	1,5	1	R	1,2
SAC.OFFIC.8276	*S. officinarum*	Malasia[b]	A	4	S	3,1	1	R	1,5
SAC.OFFIC.8280	*S. officinarum*	Malasia[b]	A	3	R	1,0	1	R	2,3
SAC.OFFIC.8284	*S. officinarum*	Malasia[b]	A	1	R	1,0	0	R	2,2
SES 205 A	*S. spontaneum*	India[a]	A	4	R	1,0	1	R	1,2
SP70-1005	BP	Brasil[a]	B3	4	S	3,6	0	R	1,5

SP70-1078	BP	Brasil[a]	B3	2	S	3,4	0	R	1,0
SP70-1143	BP	Brasil[a]	B3	2	S	3,4	0	R	1,0
SP70-1284	BP	Brasil[a]	B3	4	S	3,0	0	R	1,6
SP70-1423	BP	Brasil[a]	B3	2	S	3,4	0	R	1,5
SP70-3370	BP	Brasil[a]	B3	4	S	3,0	1	R	1,3
SP71-6163	BP	Brasil	B3	4	S	2,3	0	R	1,3
SP71-1406	BP	Brasil[a]	B3	4	S	2,4	1	R	1,0
SP71-6949	BP	Brasil[a]	B3	2	S	4,0	0	R	1,0
SP71-799	BP	Brasil[a]	B3	4	S	4,3	0	R	1,0
SP72-4928	BP	Brasil[a]	B4	2	S	4,2	0	R	1,2
SP77-5181	BP	Brasil[a]	B4	1	R	1,0	0	R	1,8
SP79-1011	BP	Brasil[a]	B4	2	S	3,4	1	R	1,0
SP79-2233	BP	Brasil[a]	B4	1	R	1,0	0	S	3,0
SP79-2312	BP	Brasil[a]	B4	1	R	1,0	1	R	1,5
SP79-2313	BP	Brasil[a]	B4	2	S	2,9	0	R	1,0
SP79-6192	BP	Brasil[a]	B4	2	S	2,0	1	R	1,0
SP80-1520	BP	Brasil[a]	B4	4	S	4,0	1	R	1,0
SP80-1816	BP	Brasil[a]	B4	1	R	1,0	0	R	1,0
SP80-1836	BP	Brasil[a]	B4	2	S	1,1	1	R	1,0
SP80-1842	BP	Brasil[a]	B4	2	S	1,3	0	R	1,0
SP80-185	BP	Brasil[a]	B4	4	S	1,7	0	R	1,0
SP80-3280	BP	Brasil[a]	B4	1	R	1,0	1	R	1,0
SP81-1763	BP	Brasil[a]	B4	1	R	1,0	0	R	1,0
SP81-3250	BP	Brasil[a]	B4	1	R	1,0	0	S	4,0
SP83-2847	BP	Brasil[a]	B5	1	R	1,0	0	R	1,4
SP83-5073	BP	Brasil[a]	B5	2	R	1,0	1	R	1,0
SP86-155	BP	Brasil[a]	B5	1	R	1,0	1	R	1,0
SP89-1115	BP	Brasil[a]	B5	1	R	1,0	0	S	4,7
SP91-1049	BP	Brasil[a]	B5	1	R	1,0	1	R	1,0
TUC71-7	BP	Argentina[a]	E	4	S	1,6	1	R	2,0

UBA DEMERARA	*S. sinense*	NA	A	4	R	1,0	0	R	1,0
US57-141-5	*S. robustum*	NA	A	3	R	1,0	0	R	1,0
US60-31-3	BP	USA[b]	E	4	S	1,1	0	R	1,1
US85-1008	*S. spontaneum x* US60-31-3	NA	A	1	R	1,0	1	R	1,0
WHITE MAURITIUS	*S. officinarum*	Índia[b]	A	4	R	1,0	1	R	1,0
WHITE PARARIA	*S. barberi*	Índia[b]	A	2	R	1,0	1	R	1,3
WHITE TRANSPARENT	*S. officinarum*	Índia[b]	A	1	R	1,0	1	R	3,0
ZWART MANILA	*S. officinarum*	Indonésia[b]	A	4	S	1,2	0	R	1,3
CTC4	BP	Brasil	B7	4	S	4,0	0	R	1,7
CTC9001	BP	Brasil	B7	1	R	1,0	1	R	1,0
RB005014	BP	Brasil	B6	1	R	1,0	0	R	1,0
RB006629	BP	Brasil	B6	1	R	1,0	1	R	2,0
RB006655	BP	Brasil	B6	1	R	1,0	1	R	1,0
RB006970	BP	Brasil	B6	1	R	1,0	0	R	1,0
RB006995	BP	Brasil	B6	1	R	1,0	0	R	1,0
RB011549	BP	Brasil	B6	1	R	1,0	1	R	1,0
RB01494	BP	Brasil	B6	3	R	1,0	0	R	1,0
RB015177	BP	Brasil	B6	3	R	1,0	0	R	1,0
RB015279	BP	Brasil	B6	1	R	1,0	1	R	1,0
RB015935	BP	Brasil	B6	1	R	1,0	1	R	1,0
RB021754	BP	Brasil	B7	4	S	5,0	0	R	1,0
RB026979	BP	Brasil	B7	1	R	1,0	1	R	1,0
RB031130	BP	Brasil	B7	1	R	1,0	1	R	1,0
RB035060	BP	Brasil	B7	4	S	2,0	1	R	1,0
RB035065	BP	Brasil	B7	2	R	1,0	0	R	1,0
RB035115	BP	Brasil	B7	1	R	1,0	0	R	1,0
RB035151	BP	Brasil	B7	1	R	1,0	0	R	1,0
RB035159	BP	Brasil	B7	1	R	1,0	1	R	1,0
RB036152	BP	Brasil	B7	1	R	1,0	1	R	1,0

RB041443	BP	Brasil	B7	4	S	3,0	1	R	1,0
RB0442	BP	Brasil	B7	4	S	3,0	1	R	1,0
RB045836	BP	Brasil	B7	2	R	1,0	1	R	1,0
RB045859	BP	Brasil	B7	1	R	1,0	1	R	2,0
RB046222	BP	Brasil	B7	1	R	1,0	0	R	1,0
RB047016	BP	Brasil	B7	1	R	1,0	0	R	1,0
RB047108	BP	Brasil	B7	3	R	1,0	0	R	2,0
RB056351	BP	Brasil	B7	1	R	1,0	1	R	1,0
RB056380	BP	Brasil	B7	4	R	1,0	1	R	1,0
RB05876	BP	Brasil	B7	2	R	1,0	0	R	1,0
RB061276	BP	Brasil	B7	4	R	1,0	0	R	3,0
RB064292	BP	Brasil	B7	4	R	1,0	1	R	1,0
RB068027	BP	Brasil	B7	1	R	1,0	0	R	1,0
RB071011	BP	Brasil	B7	1	R	1,0	1	R	3,0
RB071055	BP	Brasil	B7	2	R	1,0	0	R	1,0
RB071928	BP	Brasil	B7	4	S	6,0	0	R	1,0
RB073034	BP	Brasil	B7	1	R	1,0	0	R	1,0
RB077210	BP	Brasil	B7	1	R	1,0	0	R	1,0
RB07814	BP	Brasil	B7	1	R	1,0	1	R	2,0
RB07818	BP	Brasil	B7	1	R	1,0	1	R	1,0
RB08791	BP	Brasil	B7	4	S	2,0	1	R	1,0
RB093070	BP	Brasil	B7	4	R	1,0	0	R	1,0
RB108519	BP	Brasil	B7	1	R	1,0	0	R	1,0
RB108544	BP	Brasil	B7	2	R	1,0	0	R	1,0
RB117001	BP	Brasil	B7	4	S	6,0	0	R	1,0
RB127825	BP	Brasil	B7	3	R	1,0	0	R	1,0
RB943047	BP	Brasil	B6	4	R	1,0	1	R	1,0
RB961003	BP	Brasil	B6	1	R	1,0	0	R	1,0
RB975033	BP	Brasil	B6	1	R	1,0	0	R	1,0
RB975201	BP	Brasil	B6	1	R	1,0	0	R	1,0

RB975375	BP	Brasil	B6	1	R	1,0	0	R	1,0
RB975952	BP	Brasil	B6	1	R	1,0	0	R	1,0
RB977526	BP	Brasil	B6	1	R	1,0	0	R	1,0
RB987917	BP	Brasil	B6	1	R	1,0	0	R	1,0
RB987932	BP	Brasil	B6	3	R	1,0	1	R	2,0
RB988137	BP	Brasil	B6	3	R	1,0	1	R	1,0
RB995198	BP	Brasil	B6	3	R	1,0	0	R	1,0
