# Supplementary figures and images for: Molecular evaluation and phenotypic screening of brown and orange rust in Saccharum germplasm
Source: PLoS One. 2024 Jul 30;19(7):e0307935. doi: 10.1371/journal.pone.0307935 (PMC11288420; doi:10.1371/journal.pone.0307935)

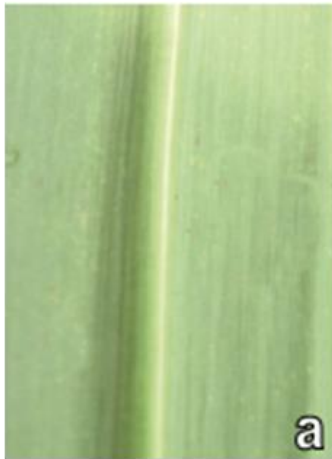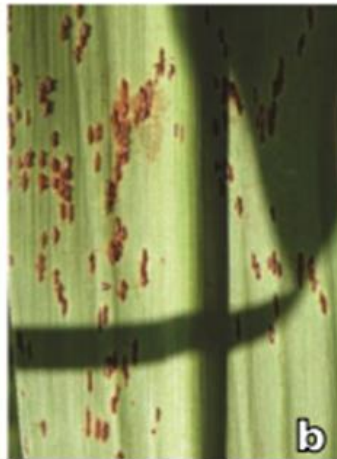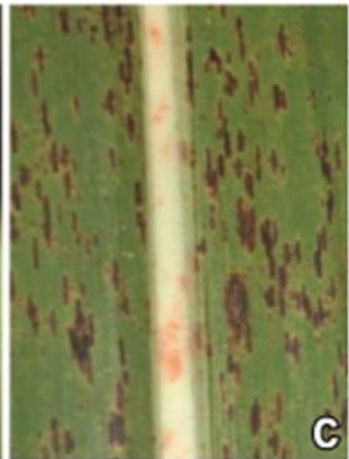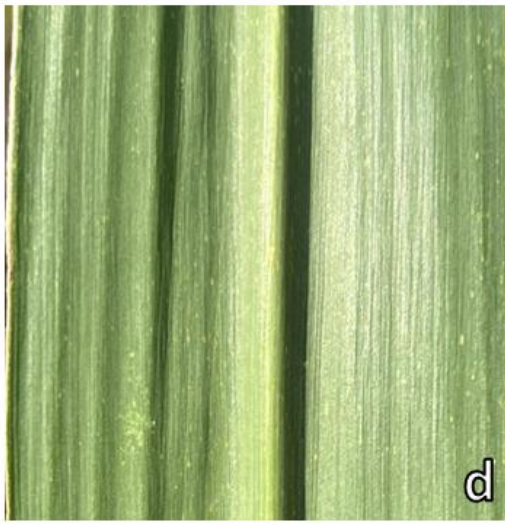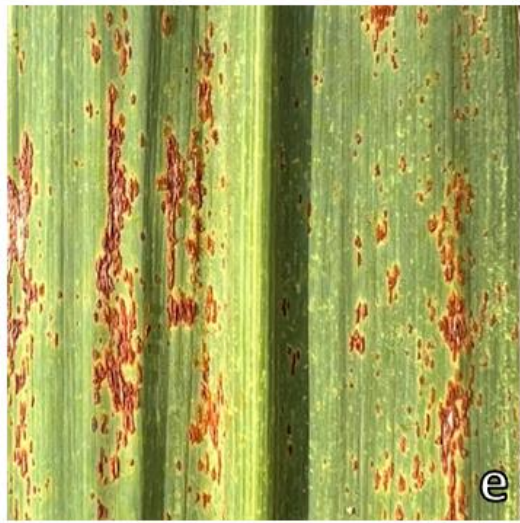

Supplement: S1 Fig — In the phenotypic evaluation, a diagrammatic scale was used with scores for disease severity (Amorim et al., [37]); S1A Fig. Leaf without disease symptom for brown rust; S1B Fig. Leaf with grade 4 on the diagrammatic scale for brown rust; S1C Fig. Leaf with a score of 6 on the diagrammatic scale for brown rust; S1D Fig. Leaf without the disease symptom for orange rust; S1E Fig. Leaf with a grade of 8 on the diagrammatic scale for orange rust. (PDF) [file pone.0307935.s002.pdf]

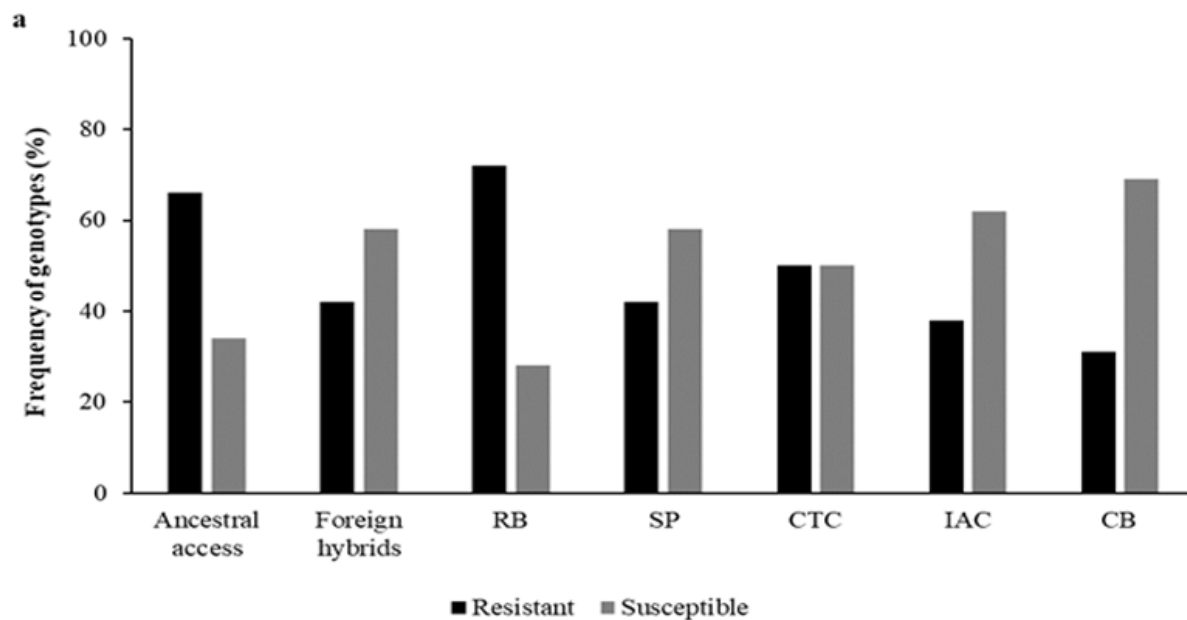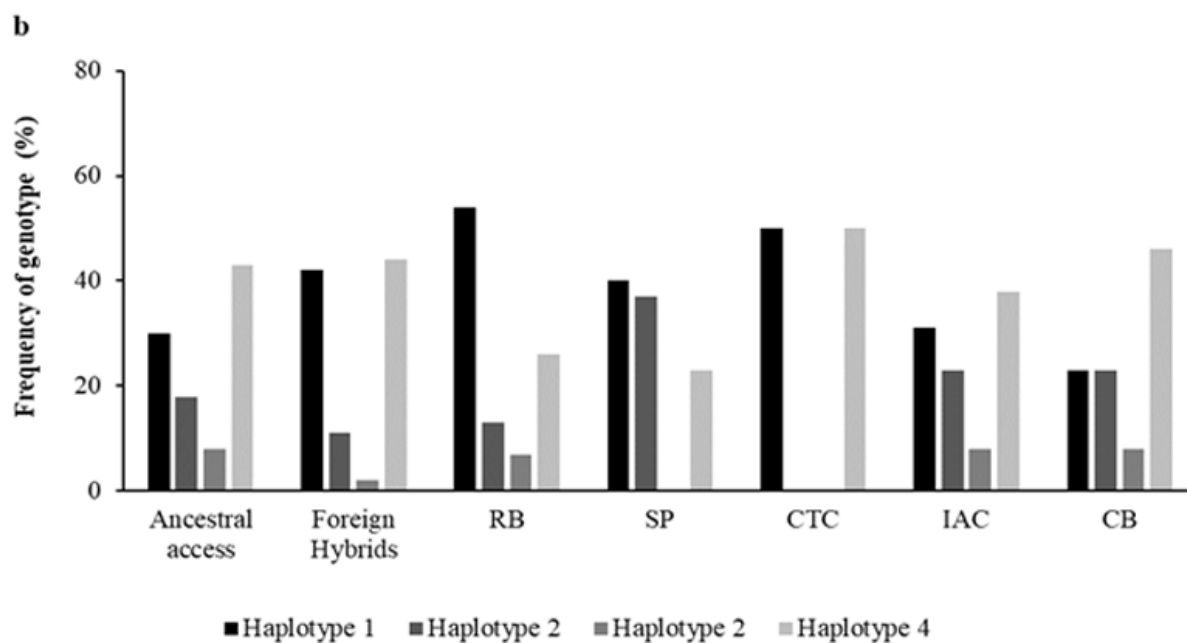

Supplement: S2 Fig — S2A Fig. Phenotypic frequency of resistant and susceptible accessions to brown rust; and S2B Fig. Frequency of Bru1 haplotypes in the nuclear collection of the 300 accessions. (PDF) [file pone.0307935.s003.pdf]

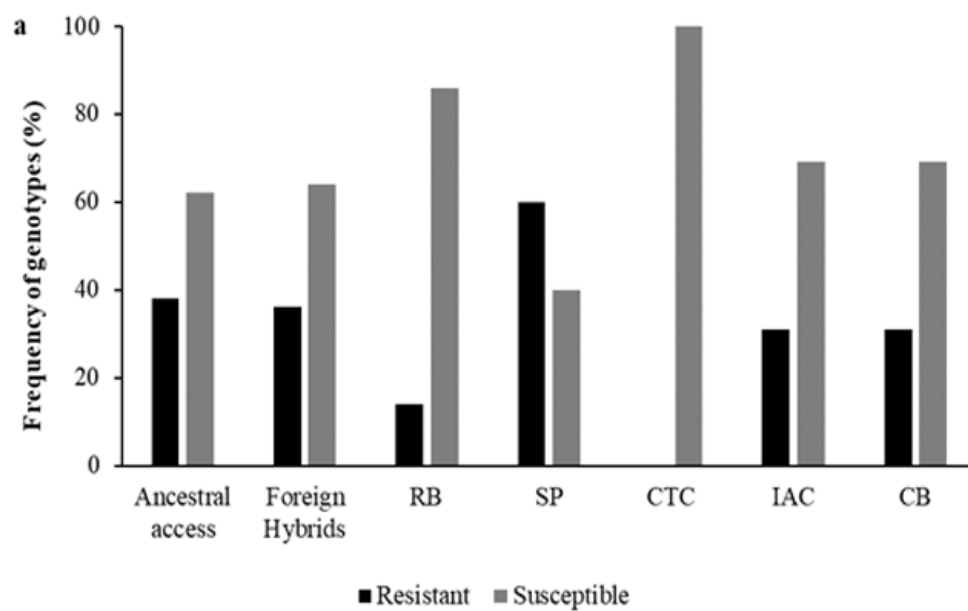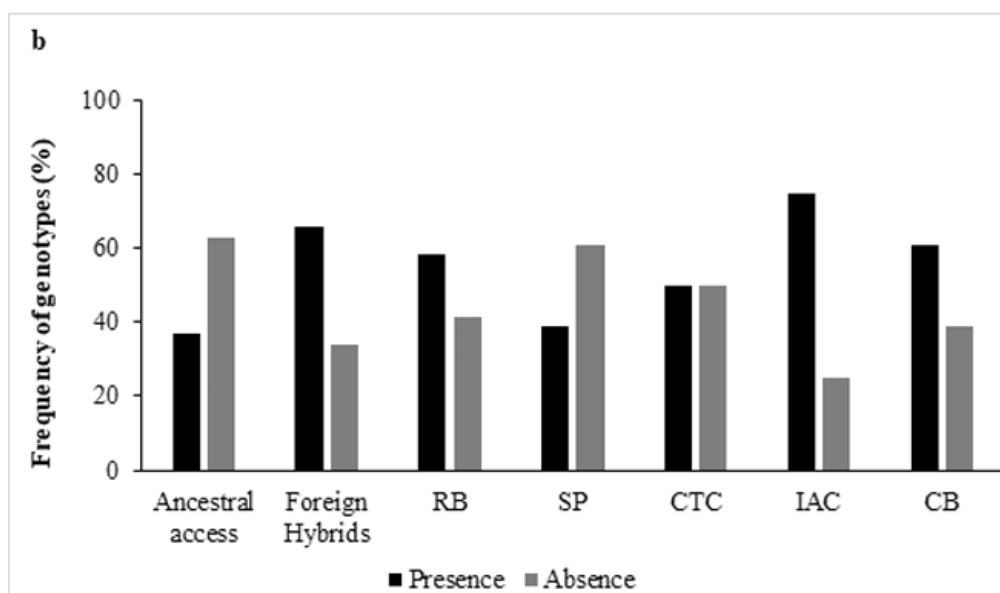

Supplement: S3 Fig — S3A Fig. Phenotypic frequency of accessions resistant and susceptible to orange rust; S3B Fig. Genotypic frequency of the presence or absence of the G1 marker in the nuclear collection of the 300 accessions. *Regarding the CP70-1547 genotype, there is no information available for the G1 marker. (PDF) [file pone.0307935.s004.pdf]

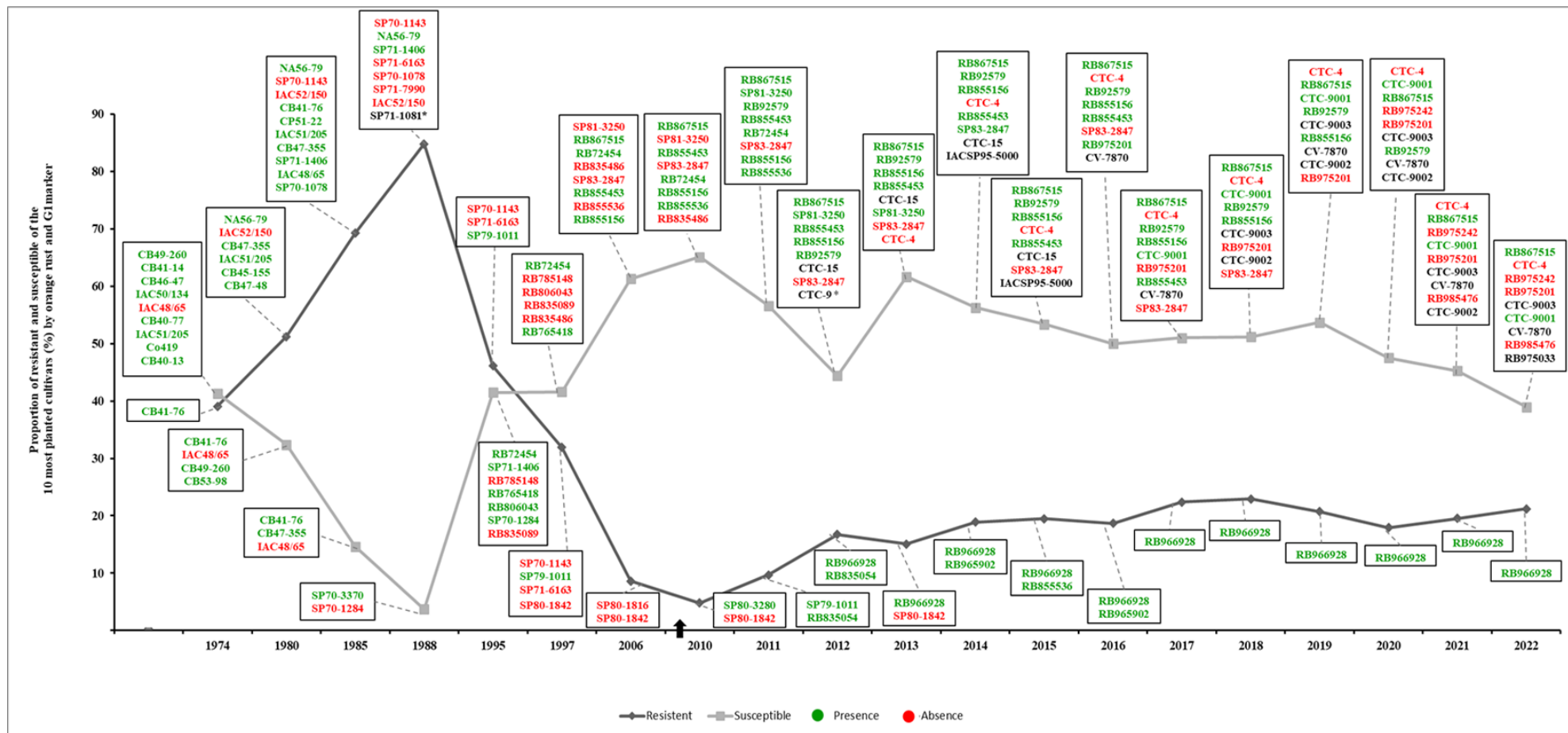

Supplement: S4 Fig — Cultivars in black do not have available information for the G1 molecular marker. (PDF) [file pone.0307935.s005.pdf]

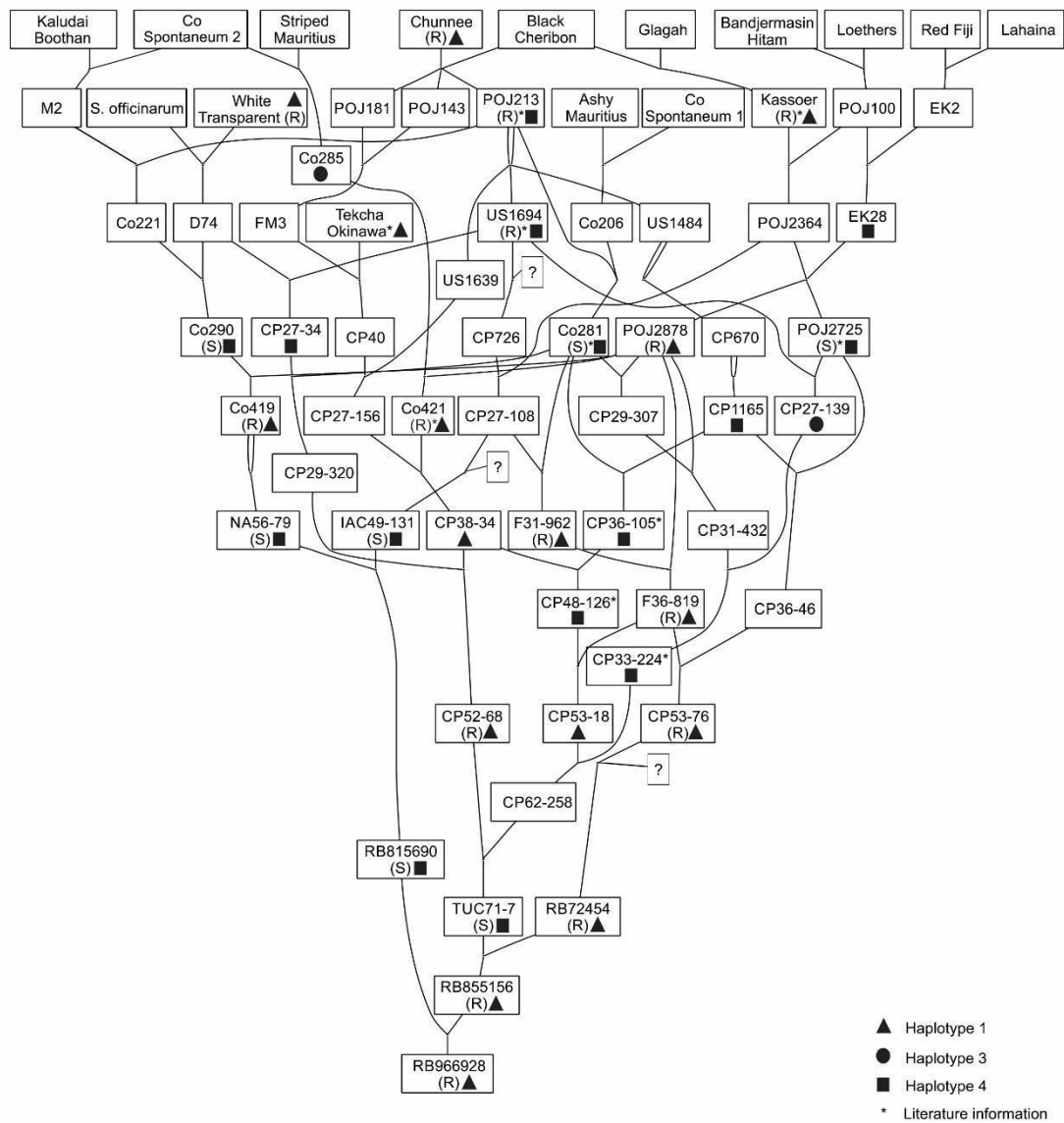

Supplement: S5 Fig — Accessions indicated with filled triangles, circles, and squares in black represent haplotypes 1, 3, and 4, respectively, for molecular markers associated with the Bru1 gene. The kinship matrix was provided by the Sugarcane Breeding Program at the Federal University of São Carlos, part of the Interuniversity Network for the Development of the Sugarcane Sector (RIDESA) (https://www.ridesaufscar.com.br/). PedigraphTM software was used to construct the genealogical tree. In the image, (R): accessions resistant to brown rust; (S): accessions susceptible to brown rust. * Information collected from the references: Costet et al., [30], Glynn et al., [56], Racedo et al., [59], Parco et al., [26], and Neuber et al., [55]. (PDF) [file pone.0307935.s006.pdf]
